# Diagnostic performance of hepatitis C virus core antigen testing for detecting hepatitis C in people living with hepatitis B: a systematic review and meta-analysis

**DOI:** 10.1186/s40249-024-01264-7

**Published:** 2024-12-02

**Authors:** Ana Treviño-Nakoura, Daniel Sepúlveda-Crespo, José M Bellon, Helena Codina, Marta Quero-Delgado, Pablo Ryan, Isidoro Martínez, Salvador Resino

**Affiliations:** 1 Servicio de Medicina Preventiva y Salud Pública, Hospital Universitario Nuestra Señora de la Candelaria, Santa Cruz de Tenerife, Spain; 2 Instituto Mixto de Investigación Escuela Nacional de Sanidad-Universidad Nacional de Educación a Distancia (IMIENS-UNED), Madrid, Spain; 3 Unidad de Infección Viral e Inmunidad, Centro Nacional de Microbiología - Instituto de Salud Carlos III, Km 2.2, 28220 Majadahonda (Madrid), Spain; 4 Centro de Investigación Biomédica en Red en Enfermedades Infecciosas (CIBERINFEC), Instituto de Salud Carlos III, Madrid, Spain; 5 Instituto de Investigación Sanitaria Gregorio Marañón (IiSGM), Madrid, Spain; 6 Servicio de Medicina Interna, Hospital Universitario Infanta Leonor, Madrid, Spain

**Keywords:** Hepatitis C, HCV core antigen, Diagnostic performance, Clinical applicability, HCV/HBV-coinfection

## Abstract

**Background:**

The current diagnostic strategy for hepatitis C virus (HCV) infection involves a two-step approach: antibody HCV screening followed by confirmatory nucleic acid testing. This study aimed to evaluate the diagnostic performance of the Abbott ARCHITECT HCV Ag assay in serum/plasma samples as a potential one-step alternative for diagnosing active HCV infection in people living with hepatitis B virus (PLWHB) through a systematic review and meta-analysis.

**Methods:**

A systematic review and meta-analysis were conducted following PRISMA-DTA guidelines. This protocol was registered on PROSPERO (CRD42023402093). A comprehensive search of electronic databases identified studies published up to 1 November 2024, comparing the ARCHITECT HCV Ag assay to an HCV-RNA reference standard. Sensitivity, specificity, and likelihood ratios were pooled using a random-effects model within the MIDAS module of Stata software. Study quality was assessed using QUADAS-2. Heterogeneity was evaluated using the Q statistic, quantified using the I², and further explored through meta-regression.

**Results:**

Ten studies (*n* = 494 participants) met inclusion criteria. The Abbott ARCHITECT HCV Ag assay demonstrated high sensitivity [91%, 95% confidence interval (*CI):* 76–97%] and specificity (99%, 95% *CI:* 99–100%). The positive likelihood ratio (PLR) was 81.20 (95% *CI:* 12.34–534.36), and the negative likelihood ratio (NLR) was 0.09 (95% *CI:* 0.03–0.27). The area under the summary receiver operating characteristic curve (AUC-SROC) was 99% (95% *CI* 98–100%). In regions with high HCV prevalence (≥ 10%), the test accurately confirmed active HCV infection in over 90% of cases. However, confirmatory testing remains necessary in low-prevalence settings (≤ 5%). The assay demonstrated an excellent ability to identify individuals without HCV infection, with a low false-negative rate (≤ 2%) regardless of HCV prevalence. Heterogeneity analysis revealed moderate to substantial variation in test performance (I² = 72.09% for sensitivity, 35.47% for PLR, and 78.33% for NLR). QUADAS-2 applicability concerns predicted heterogeneity, but differences were likely insignificant due to minimal variations and limited studies.

**Conclusions:**

The Abbott ARCHITECT HCV Ag assay exhibited promising accuracy in detecting active HCV infection among PLWHB. This test might help diagnose active HCV infection in high-prevalence scenarios (≥ 10%) but needs further confirmation in low-prevalence settings (≤ 5%).

**Graphical Abstract:**

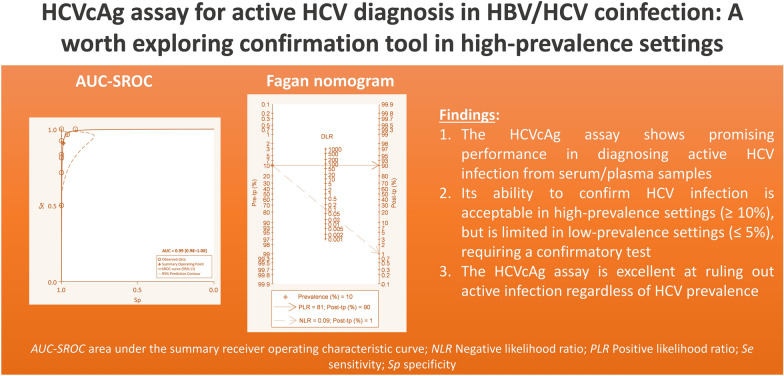

**Supplementary Information:**

The online version contains supplementary material available at 10.1186/s40249-024-01264-7.

## Background

Viral hepatitis, particularly caused by the hepatitis B virus (HBV) and hepatitis C virus (HCV), remains a significant global health burden with increasing mortality rates [1]. Chronic hepatitis, predominantly attributed to HBV and HCV, is a leading cause of liver-related deaths. HCV/HBV coinfection exacerbates disease progression, increasing the risk of cirrhosis and hepatocellular carcinoma [2]. Moreover, this coinfection complicates treatment, heightens the risk of viral reactivation and transmission, and poses a substantial challenge for infection management and control [3].

HCV/HBV coinfection is relatively common, particularly in high-risk populations such as persons who inject drugs, HIV-positive individuals, and hemodialysis patients [1]. The prevalence of hepatitis C among people living with HBV (PLWHB) varies geographically due to differing viral distributions and transmission patterns [1]. However, HCV/HBV coinfection may be underdiagnosed due to non-specific symptoms and restricted access to comprehensive screening.

HCV and HBV testing is essential to identify coinfected individuals. In particular, diagnosing active HCV infection involves two sequential steps [4]: detecting antibodies (anti-HCV) followed by directly detecting the viral genome (HCV-RNA) using polymerase chain reaction (PCR). Implementing the current HCV diagnostic algorithm in low- and middle-income countries (LMICs) and among high-risk populations poses significant challenges, resulting in low diagnostic rates and many individuals remaining unaware of their infection [5, 6]. It underscores the potential of adopting a single-stage diagnostic test to meet the WHO’s goal of diagnosing 90% of HCV-infected individuals by 2030 [7].

HCV core antigen (HCVcAg) testing offers a rapid, cost-effective, and sensitive alternative to HCV-RNA detection, capable of being performed in a single step [8]. Released into plasma during viral assembly, HCVcAg is highly conserved across various virus strains and can be detected earlier than antibodies, persisting throughout infection to indicate active HCV infection [8, 9]. Compared to the two-step algorithm, the HCVcAg test is more economical, faster (< 40 min), and allows for a sample throughput [10, 11]. Various platforms are available to detect HCVcAg [12, 13]. Among them, the Abbott ARCHITECT HCV Ag assay (Abbott Diagnostics) stands out for its widespread use and performance in sensitivity and specificity [8]. This assay has become a valuable tool for detecting active HCV infection in chronic patients and monitoring antiviral treatment [14–17]. Scientific evidence supports the clinical utility of the Abbott ARCHITECT HCV Ag assay, making it a benchmark for HCV diagnosis [8]. However, it is essential to evaluate the performance of this assay in complex clinical settings such as HCV/HBV coinfection. Therefore, consensus around assays with solid evidence, such as the Abbott ARCHITECT HCV Ag, can improve the comprehensive care of patients with viral hepatitis.

Serological tests can be more challenging to interpret in HCV/HBV coinfection due to the viruses’ interaction and the body’s immune response [3, 18]. Consequently, HCV/HBV coinfection results in lower plasma levels of HCV RNA compared to HCV monoinfection. Therefore, it is essential to consider that plasma HCVcAg levels might also be reduced, potentially affecting the diagnostic accuracy of the HCVcAg test.

This study aims to evaluate the diagnostic performance of the Abbott ARCHITECT HCV Ag assay for detecting active HCV infection in the serum/plasma of PLWHB through a systematic review and meta-analysis of all eligible studies published up to 1 November 2024.

## Methods

This systematic review adhered to rigorous methodological standards outlined in the *Cochrane Handbook for Diagnostic Test Accuracy Reviews* [19] and the Preferred Reporting Items for Systematic Reviews and Meta-analysis of Diagnostic Test Accuracy (PRISMA-DTA) statement [20]. For transparency, the PRISMA-DTA checklist is provided in Additional File 1.

### ARCHITECT HCV Ag assay

The ARCHITECT HCV Ag assay is a two-step chemiluminescent microparticle immunoassay that quantifies HCVcAg in serum or plasma samples. Microparticles coated with monoclonal anti-HCV are employed for HCVcAg detection. In the first step, sample pretreatment lyses viruses and releases HCVcAg from immune complexes. The released HCVcAg is detected in the second step. The entire process is automated on the Abbott ARCHITECT analyzer, and HCVcAg concentration is calculated using a calibration curve [13, 21].

Manufacturer’s specifications indicate a cut-off of 3 fmol/L (0.06 pg/ml), corresponding to a detection limit of approximately 500–3000 IU/ml HCV-RNA, depending on the HCV genotype [22, 23]. Results are interpreted as follows: (i) non-reactive: HCVcAg < 3 fmol/L; (ii) reactive: HCVcAg > 10 fmol/L; (iii) indeterminate: 3 fmol/L < HCVcAg < 10 fmol/L, requiring retesting. Then, a reactive result is defined as > 3 fmol/L, and a non-reactive result as < 3 fmol/L [21].

### Search strategy

We conducted a comprehensive search across multiple electronic databases, including MEDLINE/PubMed, EMBASE, SCOPUS, Web of Science, and Cochrane Library, to identify relevant studies. This search encompassed all studies published up to 1 November 2024 without language, study design, or geographic location restrictions. Our search combined terms related to hepatitis C (e.g., ‘hepatitis C’, ‘HCV’), clinical laboratory diagnoses (e.g., ‘PCR’, ‘immunoassay’), HCV antigens (e.g., ‘HCVcAg’, ‘core protein’), and the diagnostic performance of the ARCHITECT HCV Ag assay (e.g., ‘accuracy’, ‘sensitivity’). These terms were linked using the ‘AND’ operator. Additionally, we meticulously hand-searched the reference lists of included studies to identify any potential studies missed in the initial search. The search strategy, including databases, Medical Subject Heading (MeSH) terms, and keywords, is detailed in Additional File 2. Notably, this protocol was pre-registered on the International Prospective Register of Systematic Reviews (PROSPERO) [24] (registration number CRD42023402093). This rigorous approach follows best practices for reproducible, transparent, and unbiased diagnostic meta-analyses [25].

### Study selection

The following criteria were applied to ensure that studies included in this systematic review were relevant and provided high-quality data. Inclusion criteria: (i) studies evaluated the diagnostic accuracy of the Abbott ARCHITECT HCV Ag assay for detecting active HCV infection in serum, plasma, or whole-blood samples from individuals coinfected with HCV and HBV; and (ii) studies compared the Abbott ARCHITECT HCV Ag assay to a HCV-RNA test (reference method) and provided sufficient data to construct a 2 × 2 contingency table for calculating sensitivity, specificity, and other relevant statistical parameters. Exclusion criteria: (i) studies not providing original data, including reviews, meta-analyses, studies with unavailable full-text, or data published in non-research formats (e.g., chapter books, conference proceedings, editorials, case reports); (ii) publications with data that could not be extracted for the meta-analysis, even after contacting the authors; (iii) studies with very small sample sizes (*n* ≤ 8) to minimize potential bias in the random-effects model, and (iv) studies involving commercial samples, non-human subjects, or tests not commercially available.

### Data extraction

Two independent reviewers (AT-N and DS-C) initially screened and selected studies based on titles and abstracts. A third-party reviewer team (JMB and SR) verified these selections to ensure consistency. When the information in the abstract was insufficient, one reviewer (DS-C) emailed the study authors for clarification. The study was excluded if the authors could not be reached after three attempts.

### Quality assessment

To assess the methodological quality of the included studies, we employed the four key domains of the Quality Assessment of Diagnostic Accuracy Studies-2 (QUADAS-2) framework [26]: patient selection, index test, reference standard, and flow of participants, including test timing (Additional File 3). Two independent reviewers (AT-N and DS-C) evaluated each study for potential risk of bias and applicability. A third reviewer (SR) resolved any discrepancies. Risk of bias was assessed across all domains, while concerns regarding applicability focused on the first three domains (patient selection, index test, and reference standard). We categorized risk of bias and applicability ratings as ‘low,’ ‘high,’ or ‘unclear,’ with ‘unclear’ used only when insufficient data precluded a definitive judgment.

### Statistical analysis

All statistical analyses were conducted using STATA 18.0 (STATA Corp., College Station, TX, USA) with the MIDAS module and R statistical package version v4.4.1 (R Foundation for Statistical Computing, Vienna, Austria). Random-effect models accounted for potential heterogeneity across studies [19, 27]. For each included study, sensitivity, specificity, and their corresponding 95% confidence intervals (95% *CI*) were calculated using true positive (TP), false positive (FP), false negative (FN), and true negative (TN) rates derived from 2 × 2 contingency tables. A bivariate random-effects model was used to estimate pooled sensitivity, specificity, positive likelihood ratio (PLR), negative likelihood ratio (NLR), and the area under the summary receiver operating characteristic curve (AUC-SROC) for studies with both TP + FN > 0 and FP + TN > 0. A separate univariate analysis included studies with either TP + FN = 0 or FP + TN = 0.

Diagnostic accuracy was assessed using AUC-SROC values, interpreted as follows: 50–60% (low accuracy, not recommended); 60–70% (poor); 70–80% (fair); 80–90% (good); and 90–100% (excellent) [28]. The clinical validity of the HCVcAg test was assessed using three analytical tools: a likelihood ratio scatter plot, a probability modifying plot, and Fagan’s nomogram [29–31]. The likelihood ratio scatter plot visually represents the test’s diagnostic accuracy by categorizing PLR and NLR into quadrants based on their discriminative ability [29]. The probability modifying plot shows how the positive and negative predictive values (PPV and NPV) of the HCVcAg test change with varying HCV prevalence (pre-test probability) [30]. Fagan’s nomogram provides a more precise calculation of how an HCVcAg result impacts the likelihood of HCV infection (post-test probability), considering HCV prevalence [31].

Heterogeneity in effect sizes was evaluated using the Chi-squared test (Q statistic) and Higgins’ inconsistency index (I²). A *P*-value ≤ 0.10 for the Q statistic indicated statistically significant heterogeneity, suggesting the observed differences in results are unlikely due to chance alone [19]. The I² index quantified the proportion of variability not explained by sampling error, interpreted as follows: ≤ 30% (might not be important); 30–60% (moderate heterogeneity); 60–75% (substantial); and ≥ 75% (considerable) indicates a greater influence of factors other than chance on the study results [32, 33]. To understand heterogeneity sources, Galbraith’s plot (potentially revealing outliers that might influence overall results) [34], bivariate boxplots (allowing for exploration of potential covariate effects) [35], Baujat plot (highlights the individual study impact on the pooled effect and heterogeneity) [36], and meta-regression analysis (potentially identifying factors that contribute to heterogeneity) were employed [37]. Meta-regression investigated the influence of factors (*P* ≤ 0.10) [19], such as: (i) publication year (Yes ≤ 2017; No > 2017); (ii) setting (Yes LMICs; No high-income countries); (iii) sample size (Yes ≤ 50; No > 50); (iv) biological sample type (Yes serum; No plasma or serum/plasma); (v) sample condition (Yes frozen; No unknown); (vi) HCV prevalence (Yes ≤ 50; No > 50); (vii) COBAS Ampliprep/COBAS TaqMan HCV Real-time PCR assay as the reference standard (Yes/No); (viii) overall QUADAS-2 risk (Yes low/unclear; No high), (ix) QUADAS-2 risk of bias (Yes low/unclear; No high), and (x) QUADAS-2 applicability concerns (Yes low/unclear; No high).

To assess publication bias, we used two methods to obtain a more accurate and reliable estimate of the overall effect size. Deeks’ funnel plot asymmetry test was used to identify potential bias [38], with a *P* ≤ 0.10 indicating potential bias [19], while the Trim and Fill method [39] was employed to adjust for missing studies.

## Results

### Search results

**Fig. 1:**
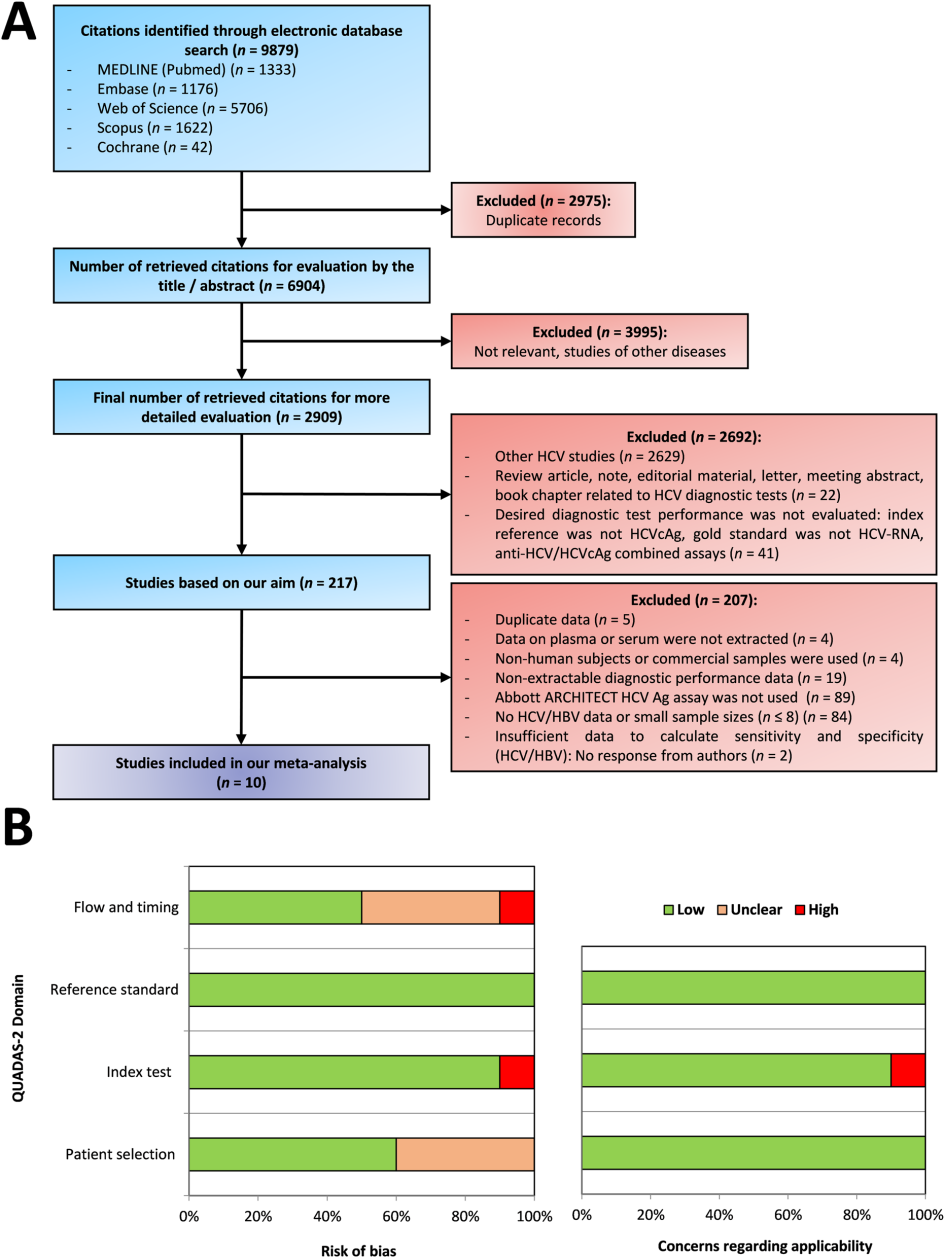
**S**earch Strategy and quality assessment. **A** presents a flow diagram outlining the search strategy used to identify relevant studies. **B** shows the QUADAS-2 risk of bias assessment for the included studies. Green indicates low risk, orange represents unclear risk, and red signifies high risk. *cAg* core antigen; *HBV* hepatitis B virus; *HCV* hepatitis C virus; *RN*A ribonucleic acid; *QUADAS* quality assessment of diagnostic accuracy study

Our search strategy identified 9879 relevant studies through a comprehensive database search (Fig. 1A). Following a rigorous review of titles and abstracts, we excluded 9662 duplicates, irrelevant records, studies unrelated to HCV, and reviews or other research articles on HCV diagnosis that did not evaluate the objective diagnostic performance. A further 207 studies were excluded from the remaining 217 potentially relevant studies because they did not mainly assess the Abbott ARCHITECT HCV Ag assay or lacked data on PLWHB. Finally, ten studies were selected for the meta-analysis [40–49].

### Article characteristics

Table 1 summarizes the key characteristics of the ten studies included in this review [40–49]. These studies were published between 2012 and 2023. While most studies employed a cross-sectional design, two were longitudinal [47, 49]. Moreover, half of the included studies were conducted in LMICs [42, 44, 45, 47, 49]. The sample size for HCVcAg detection was 494 PLWHB, with a pooled prevalence of active HCV infection of 63.06%. The average participant age across studies was 45.05 years; males comprised 72.41% of the sample.

**Table 1:** Characteristics of studies included in the meta-analysis

Author (year)	Country	*n*	Age (years)	Males (%)	HCV genotype	HCV-AVT	HIV (%)	Sample type	Sample condition	GS cut-off (IU/ml)
Mederacke et al. [40]	Germany	57	40.5	73.0	1, 2, 3, 4	NT	N/A	Serum/plasma	Frozen	15 and 600
Alonso et al. [41]	Spain	25	53.1	52.6	1, 2, 3, 4	NT/OT	75.0	Serum	Unknown	15
Duchesne et al. [42]	Cameroon	107	40.6	49.0	1, 2, 3	NT	0.0	Serum	Frozen	12
Loggi et al. [43]	Italy	35	58.0	74.2	1, 2, 3, 4	OT	0.0	Serum	Unknown	15
Mohamed et al. [44]	Tanzania	14	34.0	92.2	1, 4	NT	50.0	Serum	Frozen	15
Wasitthankasem et al. [45]	Thailand	13	45.2	69.2	1, 3, 6	NT	0.3	Plasma	Unknown	12
Alonso et al. [46]	Spain	57	N/A	N/A	1, 2, 3, 4	NT	33.3	Serum	Frozen	15
Ponnuvel et al. [47]	India	8	40.0	75.0	1, 3, 4	NT	0.0	Plasma	Frozen	12
Sun et al. [48]	Taiwan, China	154	46.0	98.1	1, 2	NT	98.1	Plasma	Unknown	15
Ponnuvel et al. [49]	India	19	48.0	68.4	1, 3, 4	NT	0.0	Plasma	Frozen	12

*All included studies evaluated populations of patients coinfected with HBV.

*N/A* Not available, *GS* Gold standard, *HBV* Hepatitis B virus, *HCV* Hepatitis C virus, *HCV-AVT* HCV Antiviral therapy, *HIV* Human immunodeficiency virus, *IU* International units, *No.* Sample size, *NT* Non-treated, *OT* On-treatment

Most studies included data from HCV treatment-naive individuals [40, 42, 44–49]; one study involved HCV therapy monitoring after treatment initiation [43], and another included a mix of treated and untreated patients [41]. HCV genotypes ranged from 1 to 6, and HIV status was reported in nine studies (prevalence ranging from 0 to 98.10%) [41–49]. Only two studies reported the inclusion of injection drug users (IDUs) [47, 48].

The Abbott ARCHITECT HCV Ag assay was used in all articles to measure HCVcAg levels through a chemiluminescence immunoassay. The gold standard method for diagnosing HCV patients was primarily the COBAS Ampliprep/COBAS TaqMan HCV (Roche Diagnostics) [41, 43, 44, 46, 48]. Four studies employed the Abbott RealTime HCV Assay (Abbott Diagnostics) [42, 45, 47, 49], while one study used a combination of COBAS Ampliprep/COBAS *Taq*Man HCV Real-time PCR (Roche Diagnostics) and Amplicor-HCV-Monitor Assay [40].

### Assessment of risk of bias

The QUADAS-2 risk assessment (detailed in Fig. 1B, Additional File 3) revealed that none of the included studies demonstrated a low risk of bias and low concerns regarding the applicability of all review questions. Patient selection remained unclear in four studies (40.0%) due to limited information on design, selection criteria, and exclusions [42, 46, 47, 49]. Moreover, one study (10%) [41] had a high risk of bias in the index test domain as the interpretation was influenced by knowledge of the reference standard results. Applicability concerns arose in another study (10%) [48] regarding the understanding and applying the HCVcAg test. Moreover, the flow and timing domain was unclear in four studies (40%) [40, 43–45] due to inadequate descriptions of the interval between tests, while another study (10%) [48] had a high risk due to poorly described intervals. All studies employed a reference standard with a low risk of bias and low concerns regarding the applicability. Given the high sensitivity and minimal variability of HCV-RNA tests, it is unlikely that knowledge of the reference standard results introduced bias into the index test.

### Diagnostic performance

**Fig. 2:**
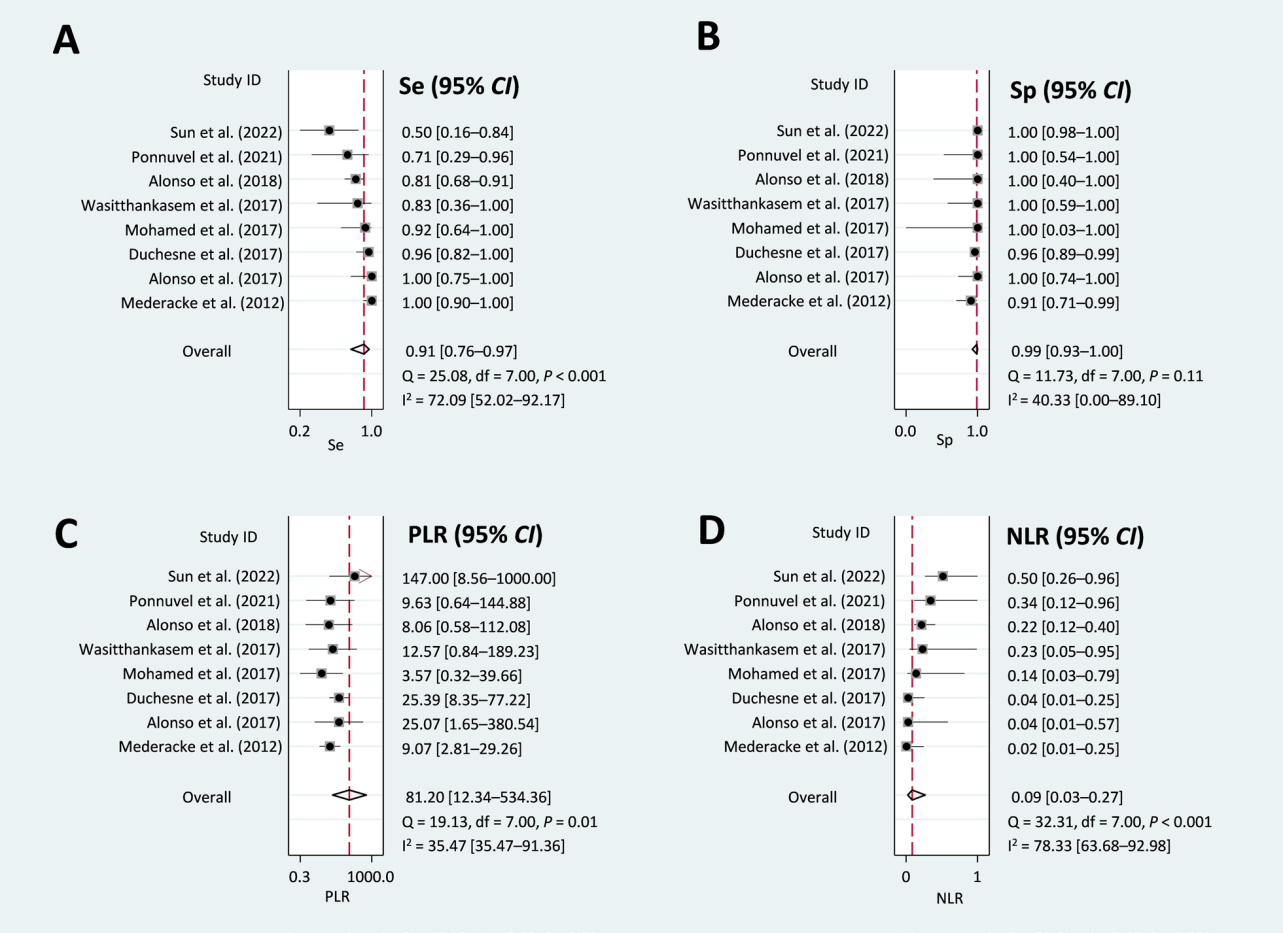
Forest plots of diagnostic accuracy measures for Abbott ARCHITECT HCV Ag assay using a bivariate random-effects model. This figure presents forest plots showing the diagnostic accuracy of the Abbott ARCHITECT HCV Ag assay for detecting active HCV infection in HCV/HBV coinfected individuals compared to a confirmatory nucleic acid test. Each panel shows **(A)** sensitivity, **(B)** specificity, **(C) **PLR, and **(D)** NLR with corresponding 95% *CI*. *95% CI* 95% confidence interval; *df* degrees of freedom; *HBV* hepatitis B virus; *HCV* hepatitis C virus; *I*^*2*^ Higgins’ inconsistency index; *NLR* negative likelihood ratio; *PLR* positive likelihood ratio; *Q* Cochran’s Q test statistic; *Se* sensitivity; *Sp* specificity

A pooled bivariate analysis of eight studies with 400 samples was conducted. Overall sensitivity was found to be 91% (95% *CI:* 76–97%) (Fig. 2A) and specificity was 99% (95% *CI:* 99–100%) (Fig. 2B). These results translate to a PLR of 81.20 (95% *CI:* 12.34–534.36) (Fig. 2C), indicating a high probability of a positive test result being TP. Conversely, the NLR was 0.09 (95% *CI:* 0.03–0.27) (Fig. 2D), suggesting that the HCVcAg test is unlikely to miss true HCV cases.

A separate pooled univariate analysis including ten studies yielded slightly higher sensitivity than the bivariate analysis (95% *CI*: 83–99%) (Additional File 4: Figure S1). Additionally, the AUC-SROC reached 99% (95% *CI*: 98–100%) (Additional File 4: Figure S2), signifying the excellent diagnostic accuracy of the HCVcAg test.

### Clinical applicability

A four-quadrant likelihood ratio scatter plot (Additional File 4: Figure S3) visually suggests that the HCVcAg test can confirm and rule out active HCV infection. Several simulations were performed using the probability modifying plot and Fagan’s plots to explore further the test’s performance across different HCV prevalence levels (0.1%, 0.5%, 1%, 5%, 10%, and 15%).

The probability modification plot (Additional File 4: Figure S4) reveals that PPV varies with HCV prevalence. For HCV prevalence ≥ 5%, PPV is reasonably high (61–80%) but decreases markedly (< 27%) at lower prevalences (≤ 1%), increasing FP risk. However, NPV remains exceptionally high (≈ 100%) across all prevalence levels, making the test highly effective for ruling out active HCV infection.

**Fig. 3:**
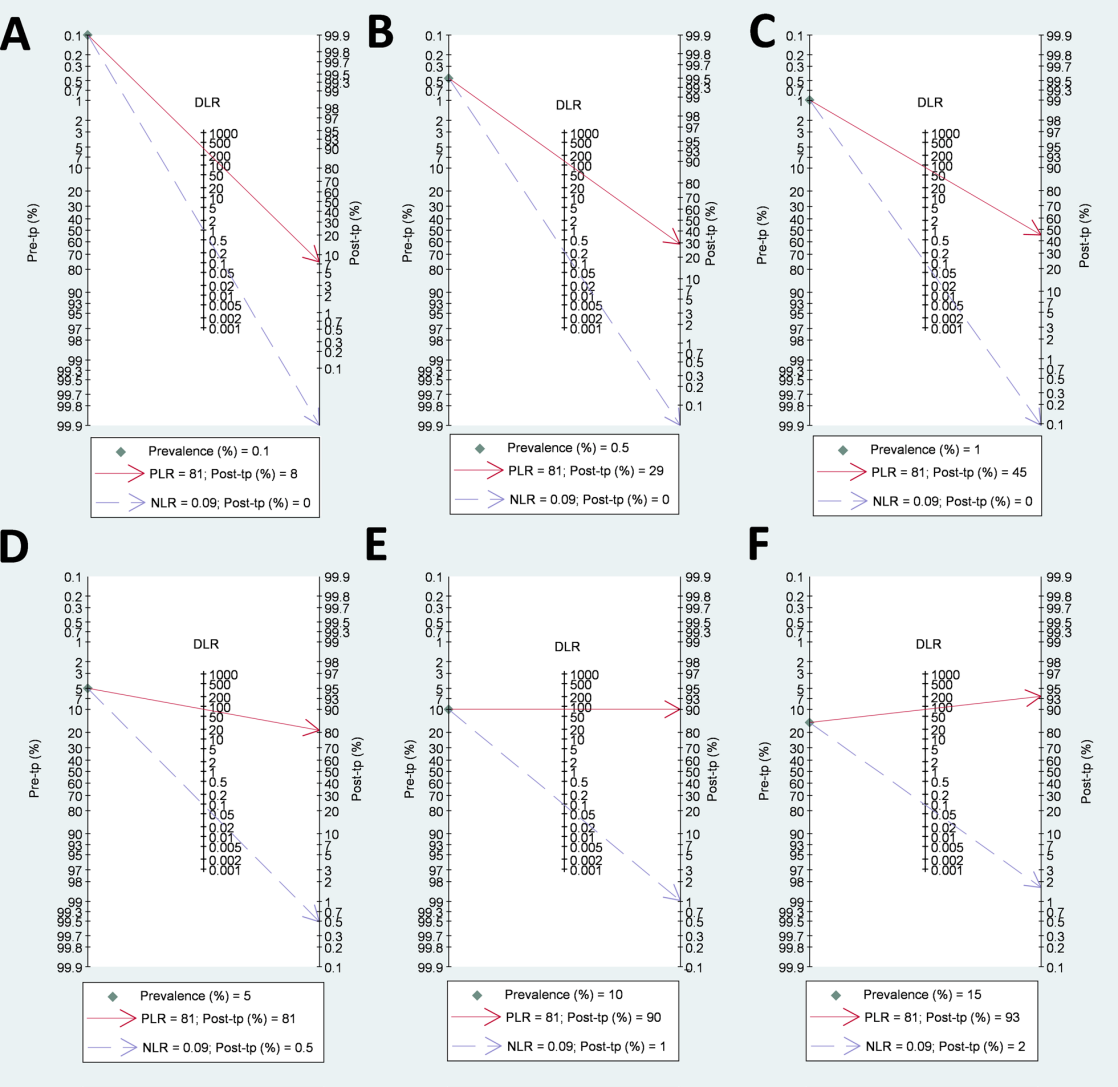
Fagan’s nomograms for Abbott ARCHITECT HCV Ag assay. This figure shows Fagan’s nomograms illustrating the post-test probability of active HCV infection in HCV/HBV coinfected individuals using the Abbott ARCHITECT HCV Ag assay compared to a confirmatory nucleic acid test at various pre-test probability levels. Each panel represents a different pre-test prevalence of HCV infection: **(A)**, 0.1% **(B)**, 0.5% **(C)**, 1% **(D)**, 5%, **(E)** 10%, and **(F)** 15%. *DLR* diagnostic likelihood ratio; *HBV* hepatitis B virus; *HCV* hepatitis C virus; *NLR* negative likelihood ratio; *PLR* positive likelihood ratio; *Post-tp* post-test probability; *Pre-tp* pre-test probability

Fagan’s plots (Fig. 3) corroborate these findings. At low prevalence (≤ 5%), the post-test probability of TP results ranges from 8 to 81%, suggesting a potential need for confirmatory testing. In contrast, at higher prevalence (≥ 10%), post-test probability exceeds 90%, reducing the need for confirmation. Notably, the likelihood of FN remains practically zero regardless of prevalence, reinforcing the test’s reliability in excluding active HCV infection.

### Exploration of heterogeneity

Heterogeneity analysis revealed moderate to substantial variation in test performance across studies. The I² for sensitivity was 72.09% (*P* < 0.001), showing considerable heterogeneity. Similarly, moderate heterogeneity was observed for PLR (I² = 35.47%, *P* < 0.001) and considerable heterogeneity for NLR (I² = 78.33%, *P* < 0.001) (Fig. 2).

Potential sources of this variation were explored visually. The Galbraith plot (Additional File 4: Figure S5A) identified one study [42] as an outlier, falling outside the 95% *CI.* Likewise, the bivariate boxplot (Additional File 4: Figure S5B) displayed outliers for three additional studies [40, 44, 48], which might contribute significantly to the overall heterogeneity. The Baujat plot (Additional File 4: Figure S6), like the Galbraith plot, identified one potential study [42] as a major contributor to heterogeneity. A sensitivity analysis was conducted to investigate the influence of individual studies further. This analysis involved excluding each study individually and examining its impact on heterogeneity and the test’s diagnostic performance parameters (Additional File 5: Table S1). Interestingly, although sensitivity analysis revealed significant variations in heterogeneity and diagnostic performance, these variations were not attributable to any particular study.

Our meta-regression analysis identified two factors significantly impacting heterogeneity (*P* ≤ 0.10) (Additional File 5: Table S2). Notably, only QUADAS-2 applicability concerns significantly influenced both sensitivity and specificity (*P* ≤ 0.10) (Additional File 5: Table S3). While other factors like year of publication, sample size, sample condition, HCV-RNA assay manufacturer, QUADAS-2 overall risk, and QUADAS-2 risk of bias showed a significant effect on specificity (*P* ≤ 0.10) (Additional File 5: Table S3), these differences are likely not clinically relevant due to minimal variations between groups and the limited number of studies included.

Finally, Deeks’ funnel plot asymmetry test (Additional File 4: Figure S7A) provided evidence suggestive of publication bias (*P* < 0.001). These findings are further supported by the results of the Trim and Fill funnel plot (Additional File 4: Figure S7B).

## Discussion

Our findings demonstrate that the Abbott ARCHITECT HCV Ag assay exhibits high accuracy (sensitivity 91%, specificity 99%) and excellent diagnostic performance (AUC-SROC 99%) in detecting active HCV infection. However, its clinical utility for confirming active HCV infection is limited, particularly in low-prevalence settings (≤ 5%) where confirmatory testing might be needed. Conversely, the assay effectively rules out active HCV infection regardless of prevalence.

To our knowledge, this is the first meta-analysis evaluating the diagnostic accuracy of the Abbott ARCHITECT HCV Ag assay for detecting active HCV infection in PLWHB serum/plasma samples. Building on our previous works demonstrating the HCVcAg test’s effectiveness in various scenarios [14–17], this study addresses the knowledge gap regarding HCV/HBV coinfection. Due to limitations in available data, a subgroup analysis of this population was not feasible. Collecting data from 10 studies encompassing 494 individuals provides a robust sample size to conclude the assay’s diagnostic accuracy in HCV/HBV coinfected patients. Our results align closely with previous meta-analyses in HCV monoinfected [16] and HCV/HIV coinfected [14] populations. However, the HCVcAg test demonstrated a higher PLR in HCV/HBV coinfected individuals than in those coinfected with HCV/HIV, possibly due to viral interactions. Moreover, viral interactions between HBV and HCV can occur in coinfected patients, leading to lower HCV RNA levels than HCV monoinfection [50], which may affect the assay’s sensitivity in patients with HCV-RNA levels below 1000 IU/ml. However, it is rare to find patients with HCV-RNA levels below 1000 IU/ml, so the impact of this interaction should be low. Future studies should investigate the effect of interactions between HBV and HCV on the diagnostic performance of the Abbott ARCHITECT HCV Ag assay.

While the Abbott Architect HCV Ag core antigen assay exhibits high sensitivity and specificity for detecting active HCV infection in individuals coinfected with HBV, it is important to note that comparability with other commercial or in-house assays is not absolute. This observation aligns with the principles outlined by Greenland [51], who highlights the variability and nuances inherent to different diagnostic methods, which the characteristics of the study population and test conditions can influence. Therefore, although the results of our meta-analysis are robust, it is essential to acknowledge that different assays may exhibit variable diagnostic performance.

Our meta-analysis strongly supports the Abbott ARCHITECT HCV Ag assay’s ability to diagnose active HCV infection, although its efficacy is influenced by HCV prevalence among PLWHB. Both the Fagan nomogram and the probability modification plot demonstrate the ability of the HCVcAg test to confirm infection decreases in populations with low prevalence, so a positive result should be interpreted with caution. In low-prevalence settings (≤ 5%), a confirmatory nucleic acid amplification test (NAAT) is recommended for positive results. Conversely, in high-prevalence settings (≥ 10%), moderately high PPV values (≥ 61%) and a high post-test probability (> 90%) of a positive result could make the HCVcAg test reasonably reliable and a potential alternative to NAATs. On the other hand, the test is excellent at ruling out HCV infection, regardless of HCV prevalence (with NPV values close to 100% and a very low FN rate [≤ 2%]).

Although the HCVcAg test’s clinical utility varies, it demonstrates robust performance across diverse HCV prevalence settings. Considering HCV prevalence is crucial for optimal test interpretation. The HCVcAg holds particular promise for marginalized populations and regions with high hepatitis C burden, where high-risk groups (IDU, homeless people, men who have sex with men, sex workers, incarcerated persons) are disproportionately affected [52]. HCV/HBV coinfection is prevalent in these populations because of shared transmission routes [1]. Moreover, the risk of HCV reinfection is heightened among vulnerable individuals due to persistent high-risk behaviors [53].

HCV/HBV coinfection prevalence varies geographically [1]. Sub-Saharan Africa (2–40%), East Asia (3–20%), Europe (3–10%), the United States (3–15%), and Latin America (2–30%) report notable disparities [1, 54, 55]. LMICs are particularly affected due to limited infection control, healthcare access, and diagnostic tools, leading to increased vulnerability among impoverished populations [52]. Early detection and treatment are essential to curb virus transmission in these settings.

The quality of included studies is crucial for meta-analysis accuracy. While no study was flawless, nine out of ten (90%) demonstrated high overall quality with minimal bias in most assessed categories. However, limitations included unclear sample selection, patient flow, and occasional missing study duration. Despite these issues, the meta-analysis’ applicability is considered high due to the low risk of bias and applicability concerns related to the reference standard. Although NAAT is not infallible, its high sensitivity and minimal variability mitigate potential biases. Ideally, only the highest quality studies would be included; however, data availability restricted this approach.

The heterogeneity of effect sizes is a critical concern in meta-analyses. We assessed heterogeneity using Cochran’s Q test and the I² statistic, providing a more reliable estimate [32]. As expected, given the complexities of diagnostic test studies, our analysis revealed substantial heterogeneity. To account for this, we employed a random-effects model [56]. Meta-regression exploring ten potential confounders identified only one with a statistically significant but clinically insignificant impact on sensitivity or specificity. While these factors were considered, other variables not included in this analysis, such as HCV viral load, subtype, HIV coinfection, RNA extraction methods, and genomic target, could also influence results.

### Study strengths

Our study employed a rigorous methodology to ensure comprehensive and unbiased literature identification. A standardized protocol was applied across multiple international biomedical databases, including studies regardless of language, design, or origin [24]. To minimize bias, article selection, data extraction, and quality assessment were independently conducted by two researchers, with a third resolving discrepancies. The search strategy encompassed a comprehensive timeframe (1976–2024) and was optimized to create a complete database, resulting in a clear and informative flow chart.

### Study limitations

Several limitations should be considered when interpreting our findings. First, the small number of included studies underscores the need for further research on the diagnostic performance of HCVcAg in PLWHB. Second, publication bias favoring positive findings may have influenced the outcomes. Third, the absence of data on HCV viral load, subtype, and sample storage conditions limited our ability to assess potential confounders. Fourth, heterogeneity in HCV-RNA extraction and detection protocols and variations in gold standards and cut-offs could introduce bias. Additionally, the manufacturer-defined HCVcAg assay cut-off hindered our ability to determine the exact HCVcAg concentration required for consistent HCV-RNA positivity. Finally, this study did not assess the cost-effectiveness of HCVcAg testing, a crucial factor for public health decision-making, especially in LMICs.

## Conclusions

The Abbott ARCHITECT HCV Ag assay demonstrated promising performance in detecting HCV infection in serum/plasma samples from PLWHB. However, its utility as a confirmatory test varied according to prevalence. In high-prevalence settings (≥ 10%), confirmation accuracy was acceptable, while in low-prevalence settings (≤ 5%), confirmatory NAAT remained essential. Moreover, it effectively ruled out active HCV infection regardless of HCV prevalence. Nevertheless, further evaluation of diagnostic accuracy in real-world clinical settings is required.

## Supplementary Information

Supplementary Material 1. Additional File 1: PRISMA-DTA Checklist and Abstracts Checklist. Additional File 2: Search strategy. Additional File 3: Risk of bias assessment adapted from QUADAS-2. Additional File 4: Figure S1. Forest plots of diagnostic accuracy measures for Abbott ARCHITECT HCV Ag assay using a univariate random-effects model. Figure S2. SROC curve plot for the Abbott ARCHITECT HCV Ag assay in detecting active HCV infection in HCV/HBV coinfected individuals compared to a confirmatory nucleic acid test. Figure S3. Likelihood ratio scatter plot for the Abbott ARCHITECT HCV Ag assay in detecting active HCV infection in HCV/HBV coinfected individuals compared to a confirmatory nucleic acid test. Figure S4. Probability modifying plot for the Abbott ARCHITECT HCV Ag assay in detecting active HCV infection in HCV/HBV coinfected individuals compared to a confirmatory nucleic acid test. Figure S5. Exploration of heterogeneity in the bivariate meta-analysis. This figure depicts two graphical tools used to explore potential sources of heterogeneity in the bivariate random-effects meta-analysis:Galbraith plot andbagplot. Figure S6. Further exploration of heterogeneity in the bivariate meta-analysis using Baujat plot. Figure S7. Deeks’ funnel plot andTrim and Fill funnel plot for publication bias in the Abbott ARCHITECT HCV Ag assay's ability to detect active HCV infection in HCV/HBV coinfected individuals, compared to a confirmatory nucleic acid test. Additional File 5: Table S1. Sensitivity analysis for diagnostic performance measures and heterogeneity. Table S2. Results of bivariate meta-regression analysis using Higgins' inconsistency indexfor subgroup analysis of the Abbott ARCHITECT HCV Ag assay in detecting active HCV infection in HCV/HBV coinfected individuals compared to a confirmatory nucleic acid test. Table S3. Results of bivariate meta-regression analysis using sensitivity and specificity for subgroup analysis of the Abbott ARCHITECT HCV Ag assay in detecting active HCV infection in HCV/HBV coinfected individuals compared to a confirmatory nucleic acid test

## Data Availability

All data generated or analyzed during this study are included in this published article and its supplementary information files.
