# Supplementary material for: Diagnostic performance of hepatitis C virus core antigen testing for detecting hepatitis C in people living with hepatitis B: a systematic review and meta-analysis
Source: Infect Dis Poverty. 2024 Dec 2;13:89. doi: 10.1186/s40249-024-01264-7 (PMC11610273; doi:10.1186/s40249-024-01264-7)
Supplement: Supplementary file 1 — Supplementary Material 1. Additional File 1: PRISMA-DTA Checklist and Abstracts Checklist. Additional File 2: Search strategy. Additional File 3: Risk of bias assessment adapted from QUADAS-2. Additional File 4: Figure S1. Forest plots of diagnostic accuracy measures for Abbott ARCHITECT HCV Ag assay using a univariate random-effects model. Figure S2. SROC curve plot for the Abbott ARCHITECT HCV Ag assay in detecting active HCV infection in HCV/HBV coinfected individuals compared to a confirmatory nucleic acid test. Figure S3. Likelihood ratio scatter plot for the Abbott ARCHITECT HCV Ag assay in detecting active HCV infection in HCV/HBV coinfected individuals compared to a confirmatory nucleic acid test. Figure S4. Probability modifying plot for the Abbott ARCHITECT HCV Ag assay in detecting active HCV infection in HCV/HBV coinfected individuals compared to a confirmatory nucleic acid test. Figure S5. Exploration of heterogeneity in the bivariate meta-analysis. This figure depicts two graphical tools used to explore potential sources of heterogeneity in the bivariate random-effects meta-analysis:Galbraith plot andbagplot. Figure S6. Further exploration of heterogeneity in the bivariate meta-analysis using Baujat plot. Figure S7. Deeks’ funnel plot andTrim and Fill funnel plot for publication bias in the Abbott ARCHITECT HCV Ag assay's ability to detect active HCV infection in HCV/HBV coinfected individuals, compared to a confirmatory nucleic acid test. Additional File 5: Table S1. Sensitivity analysis for diagnostic performance measures and heterogeneity. Table S2. Results of bivariate meta-regression analysis using Higgins' inconsistency indexfor subgroup analysis of the Abbott ARCHITECT HCV Ag assay in detecting active HCV infection in HCV/HBV coinfected individuals compared to a confirmatory nucleic acid test. Table S3. Results of bivariate meta-regression analysis using sensitivity and specificity for subgroup analysis of the Abbott ARCHITECT HCV Ag assay in de [file 40249_2024_1264_MOESM1_ESM.docx]

# Supplementary material

**Title**: Diagnostic performance of hepatitis c virus core antigen testing for detecting hepatitis C in people living with hepatitis B: a systematic review and meta-analysis

**Authors**: Ana TREVIÑO-NAKOURA ^1,2,†^; Daniel SEPÚLVEDA-CRESPO ^3,4,†,*,^; José M BELLON ^5^; Helena CODINA ^3^; Marta QUERO-DELGADO ^3^; Pablo RYAN ^4,6^; Isidoro MARTÍNEZ ^3,4,¥^; Salvador RESINO ^3,4,¥,*^

**Current affiliations**:

(1) Servicio de Medicina Preventiva y Salud Pública, Hospital Universitario Nuestra Señora de la Candelaria, Santa Cruz de Tenerife, Spain.

(2) Instituto Mixto de Investigación Escuela Nacional de Sanidad-Universidad Nacional de Educación a Distancia (IMIENS-UNED), Madrid, Spain

(3) Unidad de Infección Viral e Inmunidad. Centro Nacional de Microbiología - Instituto de Salud Carlos III, Majadahonda, Spain.

(4) Centro de Investigación Biomédica en Red en Enfermedades Infecciosas (CIBERINFEC), Instituto de Salud Carlos III, Madrid, Spain.

(5) Instituto de Investigación Sanitaria Gregorio Marañón (IiSGM), Madrid, Spain.

(6) Servicio de Medicina Interna, Hospital Universitario Infanta Leonor, Madrid, Spain.

#
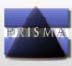
Additional file 1: PRISMA-DTA Checklist and Abstracts Checklist

| **Section and Topic** | **Item #** | **Checklist item** | **Location where item is reported** |
| --- | --- | --- | --- |
| **TITLE** | | |  |
| Title | 1 | Identify the report as a systematic review. | 1 |
| **ABSTRACT** | | |  |
| Abstract | 2 | See the PRISMA 2020 for Abstracts checklist. | 3-4 |
| **INTRODUCTION** | | |  |
| Rationale | 3 | Describe the rationale for the review in the context of existing knowledge. | 5-6 |
| Objectives | 4 | Provide an explicit statement of the objective(s) or question(s) the review addresses. | 6 |
| **METHODS** | | |  |
| Eligibility criteria | 5 | Specify the inclusion and exclusion criteria for the review and how studies were grouped for the syntheses. | 7 |
| Information sources | 6 | Specify all databases, registers, websites, organisations, reference lists and other sources searched or consulted to identify studies. Specify the date when each source was last searched or consulted. | 4-7 |
| Search strategy | 7 | Present the full search strategies for all databases, registers and websites, including any filters and limits used. | Suppl. File 2 |
| Selection process | 8 | Specify the methods used to decide whether a study met the inclusion criteria of the review, including how many reviewers screened each record and each report retrieved, whether they worked independently, and if applicable, details of automation tools used in the process. | 6-7 |
| Data collection process | 9 | Specify the methods used to collect data from reports, including how many reviewers collected data from each report, whether they worked independently, any processes for obtaining or confirming data from study investigators, and if applicable, details of automation tools used in the process. | 6-7 |
| Data items | 10a | List and define all outcomes for which data were sought. Specify whether all results that were compatible with each outcome domain in each study were sought (e.g. for all measures, time points, analyses), and if not, the methods used to decide which results to collect. | 7-8 |
|  | 10b | List and define all other variables for which data were sought (e.g. participant and intervention characteristics, funding sources). Describe any assumptions made about any missing or unclear information. | 7-8 |
| Study risk of bias assessment | 11 | Specify the methods used to assess risk of bias in the included studies, including details of the tool(s) used, how many reviewers assessed each study and whether they worked independently, and if applicable, details of automation tools used in the process. | 7 |
| Effect measures | 12 | Specify for each outcome the effect measure(s) (e.g. risk ratio, mean difference) used in the synthesis or presentation of results. | 6-8 |
| Synthesis methods | 13a | Describe the processes used to decide which studies were eligible for each synthesis (e.g. tabulating the study intervention characteristics and comparing against the planned groups for each synthesis (item #5)). | 6-8 |
|  | 13b | Describe any methods required to prepare the data for presentation or synthesis, such as handling of missing summary statistics, or data conversions. | 6-8 |
|  | 13c | Describe any methods used to tabulate or visually display results of individual studies and syntheses. | 6-8 |
|  | 13d | Describe any methods used to synthesize results and provide a rationale for the choice(s). If meta-analysis was performed, describe the model(s), method(s) to identify the presence and extent of statistical heterogeneity, and software package(s) used. | 6-8 |
|  | 13e | Describe any methods used to explore possible causes of heterogeneity among study results (e.g. subgroup analysis, meta-regression). | 8 |
|  | 13f | Describe any sensitivity analyses conducted to assess robustness of the synthesized results. | 8 |
| Reporting bias assessment | 14 | Describe any methods used to assess risk of bias due to missing results in a synthesis (arising from reporting biases). | 7-8 |
| Certainty assessment | 15 | Describe any methods used to assess certainty (or confidence) in the body of evidence for an outcome. | 7-8 |
| **RESULTS** | | |  |
| Study selection | 16a | Describe the results of the search and selection process, from the number of records identified in the search to the number of studies included in the review, ideally using a flow diagram. | 9 |
|  | 16b | Cite studies that might appear to meet the inclusion criteria, but which were excluded, and explain why they were excluded. | 9 |
| Study characteristics | 17 | Cite each included study and present its characteristics. | Table 1 |
| Risk of bias in studies | 18 | Present assessments of risk of bias for each included study. | 9-10 |
| Results of individual studies | 19 | For all outcomes, present, for each study: (a) summary statistics for each group (where appropriate) and (b) an effect estimate and its precision (e.g. confidence/credible interval), ideally using structured tables or plots. | Fig. 2 and Suppl. Fig. 1 |
| Results of syntheses | 20a | For each synthesis, briefly summarise the characteristics and risk of bias among contributing studies. | 9-10 |
|  | 20b | Present results of all statistical syntheses conducted. If meta-analysis was done, present for each the summary estimate and its precision (e.g. confidence/credible interval) and measures of statistical heterogeneity. If comparing groups, describe the direction of the effect. | 10-11 |
|  | 20c | Present results of all investigations of possible causes of heterogeneity among study results. | 10-11 |
|  | 20d | Present results of all sensitivity analyses conducted to assess the robustness of the synthesized results. | 10-11 |
| Reporting biases | 21 | Present assessments of risk of bias due to missing results (arising from reporting biases) for each synthesis assessed. | 11 |
| Certainty of evidence | 22 | Present assessments of certainty (or confidence) in the body of evidence for each outcome assessed. | 11 |
| **DISCUSSION** | | |  |
| Discussion | 23a | Provide a general interpretation of the results in the context of other evidence. | 11 |
|  | 23b | Discuss any limitations of the evidence included in the review. | 13 |
|  | 23c | Discuss any limitations of the review processes used. | 13 |
|  | 23d | Discuss implications of the results for practice, policy, and future research. | 11-14 |
| **OTHER INFORMATION** | | |  |
| Registration and protocol | 24a | Provide registration information for the review, including register name and registration number, or state that the review was not registered. | 6-7 |
|  | 24b | Indicate where the review protocol can be accessed, or state that a protocol was not prepared. | 6-7 |
|  | 24c | Describe and explain any amendments to information provided at registration or in the protocol. | 6-7 |
| Support | 25 | Describe sources of financial or non-financial support for the review, and the role of the funders or sponsors in the review. | 15 |
| Competing interests | 26 | Declare any competing interests of review authors. | 15 |
| Availability of data, code and other materials | 27 | Report which of the following are publicly available and where they can be found: template data collection forms; data extracted from included studies; data used for all analyses; analytic code; any other materials used in the review. | 15 |

| **Topic** | **No.** | **Item for Abstracts** | **Reported?** |
| --- | --- | --- | --- |
| **TITLE** |  |  |  |
| **Title** | 1 | Identify the report as a systematic review. | Yes |
| **BACKGROUND** |  |  |  |
| **Objectives** | 2 | Provide an explicit statement of the main objective(s) or question(s) the review addresses. | Yes |
| **METHODS** |  |  |  |
| **Eligibility criteria** | 3 | Specify the inclusion and exclusion criteria for the review. | Yes |
| **Information sources** | 4 | Specify the information sources (e.g. databases, registers) used to identify studies and the date when each was last searched. | Yes |
| **Risk of bias** | 5 | Specify the methods used to assess risk of bias in the included studies. | Yes |
| **Synthesis of results** | 6 | Specify the methods used to present and synthesize results. | Yes |
| **RESULTS** |  |  |  |
| **Included studies** | 7 | Give the total number of included studies and participants and summarise relevant characteristics of studies. | Yes |
| **Synthesis of results** | 8 | Present results for main outcomes, preferably indicating the number of included studies and participants for each. If meta-analysis was done, report the summary estimate and confidence/credible interval. If comparing groups, indicate the direction of the effect (i.e. which group is favoured). | Yes |
| **DISCUSSION** |  |  |  |
| **Limitations of evidence** | 9 | Provide a brief summary of the limitations of the evidence included in the review (e.g. study risk of bias, inconsistency and imprecision). | Yes |
| **Interpretation** | 10 | Provide a general interpretation of the results and important implications. | Yes |
| **OTHER** |  |  |  |
| **Funding** | 11 | Specify the primary source of funding for the review. | No |
| **Registration** | 12 | Provide the register name and registration number. | Yes |

#
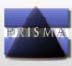
Additional file 2: Search strategy

## **Search strategy PubMed**

("hepatitis c"[MeSH Terms] OR "hepacivirus"[MeSH Terms] OR ("hepatitis c"[Title/Abstract] OR "hepatitis c virus"[Title/Abstract] OR "hepatitis c viruses"[Title/Abstract] OR "hepatitis c like virus"[Title/Abstract] OR "hepatitis c like viruses"[Title/Abstract] OR "hepatitis virus type c"[Title/Abstract] OR "hcv"[Title/Abstract] OR "h c v"[Title/Abstract] OR "vhc"[Title/Abstract] OR "v h c"[Title/Abstract] OR "hepacivirus"[Title/Abstract] OR "hepaciviruses"[Title/Abstract] OR "hcv viral"[Title/Abstract] OR "hcv infected"[Title/Abstract] OR "hcv infection"[Title/Abstract] OR "hcv rna"[Title/Abstract] OR "hepatitis c virus rna"[Title/Abstract] OR "parenterally transmitted non a non"[Title/Abstract] OR "pt nanbh"[Title/Abstract])) AND ("diagnosis"[MeSH Terms] OR "diagnostic techniques and procedures"[MeSH Terms] OR "clinical laboratory techniques"[MeSH Terms] OR "mass screening"[MeSH Terms] OR "nucleic acid amplification techniques"[MeSH Terms] OR "rna"[MeSH Terms] OR "rna, viral/blood"[MeSH Terms] OR ("clinical laboratory diagnoses"[Title/Abstract] OR "clinical laboratory diagnostic"[Title/Abstract] OR "clinical laboratory techniques"[Title/Abstract] OR "clinical laboratory testing"[Title/Abstract] OR "diagnose"[Title/Abstract] OR "diagnoses"[Title/Abstract] OR "diagnosis of hcv"[Title/Abstract] OR "diagnosis"[Title/Abstract] OR "diagnostic techniques and procedures"[Title/Abstract]OR "diagnostic"[Title/Abstract] OR "hcv infection diagnosis"[Title/Abstract] OR "hcv testing"[Title/Abstract] OR "mass screening"[Title/Abstract] OR "mass screenings"[Title/Abstract] OR "molecular diagnostic techniques"[Title/Abstract] OR "screening approach"[Title/Abstract] OR "screening"[Title/Abstract] OR "testing diagnostic"[Title/Abstract] OR "plasma levels"[Title/Abstract] OR "sera"[Title/Abstract] OR "serum levels"[Title/Abstract] OR "dried blood filter” [Title/Abstract] OR "dried blood spot"[Title/Abstract] OR "dried blood” [Title/Abstract] OR "dried sample” [Title/Abstract] OR "filter paper” [Title/Abstract] OR "Whatman” [Title/Abstract] OR “DBS” [Title/Abstract] OR "assay kits"[Title/Abstract] OR "hcv assays"[Title/Abstract] OR "hcv pcr assay"[Title/Abstract] OR "hcv pcr method"[Title/Abstract] OR "hcv pcr"[Title/Abstract] OR "hcv rna"[Title/Abstract] OR "hcv rna levels"[Title/Abstract] OR "hcv rna quantification"[Title/Abstract] OR "hcv rna quantification assays"[Title/Abstract] OR "hepatitis c markers"[Title/Abstract] OR "hepatitis markers"[Title/Abstract] OR "immunoassay"[Title/Abstract] OR "quantitative assays"[Title/Abstract] OR "quantitative reverse transcription pcr"[Title/Abstract] OR "real time pcr"[Title/Abstract] OR "rna levels"[Title/Abstract] OR "roche cobas taqman assays"[Title/Abstract] OR "roche cobas taqman hcv"[Title/Abstract])) AND ("hepatitis c antigens"[MeSH Terms] OR ("antigens"[Title/Abstract] OR "core antigen assay"[Title/Abstract] OR "core antigen assays"[Title/Abstract] OR "core antigen test"[Title/Abstract] OR "core antigen"[Title/Abstract] OR "hcv ag assay"[Title/Abstract] OR "hcv ag detection"[Title/Abstract]OR "hcv ag"[Title/Abstract] OR "hcv antigen testing"[Title/Abstract] OR "hcv antigen"[Title/Abstract] OR "HCVcAg"[Title/Abstract] OR "hcv core antigen assay"[Title/Abstract] OR "hcv core antigen assays"[Title/Abstract] OR "hcv core antigen detection"[Title/Abstract] OR "hcv core antigen determination"[Title/Abstract] OR "hcv core antigen testing"[Title/Abstract] OR "hcv core antigen"[Title/Abstract] OR "hcv core protein"[Title/Abstract] OR "hcv core region"[Title/Abstract] OR "hcv cp"[Title/Abstract] OR "hcvcoreag"[Title/Abstract] OR "hepatitis c antigens"[Title/Abstract] OR "hepatitis c virus core antigen"[Title/Abstract] OR "hepatitis c virus core"[Title/Abstract] OR "hepatitis non a non b antigen"[Title/Abstract] OR "viral core proteins"[Title/Abstract])) AND ("accuracy"[Title/Abstract] OR "correlation"[Title/Abstract] OR "correlations"[Title/Abstract] OR "negative predictive power"[Title/Abstract] OR "negative predictive value"[Title/Abstract] OR "negative predictive values"[Title/Abstract] OR "NPV"[Title/Abstract] OR "positive predictive power"[Title/Abstract] OR "positive predictive value"[Title/Abstract] OR "positive predictive values"[Title/Abstract] OR "PPV"[Title/Abstract] OR "receiver operating characteristics"[Title/Abstract] OR "regression analysis"[Title/Abstract] OR "ROC"[Title/Abstract] OR "sensitive"[Title/Abstract] OR "sensitivities"[Title/Abstract] OR "sensitivity"[Title/Abstract] OR "specific"[Title/Abstract] OR "specificity"[Title/Abstract] OR “Abbott ARCHITECT HCV Ag assay” OR “Abbott ARCHITECT HCV Ag test” OR “Abbott ARCHITECT i2000SR” OR “Abbott ARCHITECT test” OR “Abbott Diagnostics” OR “Abbott HCV core antigen” OR “Abbott Laboratories” OR “ARCHITECT” OR “ARCHITECT ci8200” OR “Architect core antigen” OR “Architect HCV Ag” OR “ARCHITECT HCV Core antigen” OR “ARCHITECT i2000SR” OR “ARCHITECT system” OR "cleia method" OR “chemiluminescence immunoassay”) NOT ("review"[Publication Type]) NOT ("meta-analysis"[Publication Type]) NOT ("systematic review"[Publication Type])

## **Search strategy Embase**

#1 'hepatitis c'/exp OR 'hepacivirus'/exp OR 'hepatitis c virus':ti,ab,kw OR 'hepatitis c viruses':ti,ab,kw OR 'hepatitis c like viruses':ti,ab,kw OR 'hepatitis virus type c':ti,ab,kw OR hcv:ti,ab,kw OR 'h c v':ti,ab,kw OR vhc:ti,ab,kw OR 'v h c':ti,ab,kw OR hepacivirus:ti,ab,kw OR hepaciviruses:ti,ab,kw OR 'parenterally transmitted non a non':ti,ab,kw

#2 'diagnosis'/exp OR ('diagnostic techniques'/exp AND 'procedures'/exp) OR 'clinical laboratory techniques'/exp OR 'nucleic acid amplification techniques'/exp OR 'rna'/exp OR 'clinical laboratory diagnostic':ti,ab,kw OR 'clinical laboratory techniques':ti,ab,kw OR 'clinical laboratory testing':ti,ab,kw OR diagnose:ti,ab,kw OR diagnoses:ti,ab,kw OR 'diagnosis of hcv':ti,ab,kw OR diagnosis:ti,ab,kw OR ('diagnostic techniques':ti,ab,kw AND procedures:ti,ab,kw) OR diagnostic:ti,ab,kw OR 'hcv testing':ti,ab,kw OR 'mass screening':ti,ab,kw OR 'mass screenings':ti,ab,kw OR 'screening approach':ti,ab,kw OR screening:ti,ab,kw OR 'plasma levels':ti,ab,kw OR sera:ti,ab,kw OR 'serum levels':ti,ab,kw OR 'dried blood filter':ti,ab,kw OR 'dried blood spot':ti,ab,kw OR 'dried blood':ti,ab,kw OR 'dried sample':ti,ab,kw OR 'filter paper':ti,ab,kw OR whatman:ti,ab,kw OR dbs:ti,ab,kw OR 'assay kits':ti,ab,kw OR 'hcv assays':ti,ab,kw OR 'hcv pcr assay':ti,ab,kw OR 'hcv pcr':ti,ab,kw OR 'hcv rna levels':ti,ab,kw OR 'hcv rna quantification':ti,ab,kw OR 'hepatitis c markers':ti,ab,kw OR 'hepatitis markers':ti,ab,kw OR immunoassay:ti,ab,kw OR 'quantitative assays':ti,ab,kw OR 'quantitative reverse transcription pcr':ti,ab,kw OR 'real time pcr':ti,ab,kw OR 'rna levels':ti,ab,kw OR 'roche cobas taqman':ti,ab,kw

#3 'hepatitis c antigens'/exp OR antigens:ti,ab,kw OR 'cleia method':ti,ab,kw OR 'core antigen assay':ti,ab,kw OR 'core antigen assays':ti,ab,kw OR 'core antigen test':ti,ab,kw OR 'core antigen':ti,ab,kw OR 'hcv ag assay':ti,ab,kw OR 'hcv ag detection':ti,ab,kw OR 'hcv ag':ti,ab,kw OR 'hcv antigen testing':ti,ab,kw OR 'hcv antigen':ti,ab,kw OR hcvcoreag:ti,ab,kw OR hcvcag:ti,ab,kw OR 'hepatitis non a non b antigen':ti,ab,kw OR 'viral core proteins':ti,ab,kw

#4 'accuracy':ti,ab,kw OR 'correlation':ti,ab,kw OR 'correlations':ti,ab,kw OR 'negative predictive power':ti,ab,kw OR 'negative predictive value':ti,ab,kw OR 'negative predictive values':ti,ab,kw OR 'NPV':ti,ab,kw OR 'positive predictive power':ti,ab,kw OR 'positive predictive value':ti,ab,kw OR 'positive predictive values':ti,ab,kw OR 'PPV':ti,ab,kw OR 'receiver operating characteristics':ti,ab,kw OR 'regression analysis':ti,ab,kw OR 'ROC':ti,ab,kw OR 'sensitive':ti,ab,kw OR 'sensitivities':ti,ab,kw OR 'sensitivity':ti,ab,kw OR 'specific':ti,ab,kw OR 'specificity':ti,ab,kw OR 'Abbott ARCHITECT HCV Ag assay' OR ' Abbott ARCHITECT HCV Ag test' OR 'Abbott ARCHITECT i2000SR' OR 'Abbott ARCHITECT test' OR 'Abbott Diagnostics' OR 'Abbott HCV Ag' OR 'Abbott HCV core antigen' OR 'Abbott Laboratories' OR 'ARCHITECT' OR 'Architect core antigen' OR 'ARCHITECT i2000SR'

#5 #1 AND #2 AND #3 AND #4

#6 #5 AND ('Article'/it OR 'Article in Press'/it)

## **Search strategy Web of Knowledge (WoS)**

#1 ((((((((((((((((((TS=(hepatitis c)) OR TS=(hepatitis c virus)) OR TS=(hepatitis c viruses)) OR TS=(hepatitis c like virus)) OR TS=(hepatitis c like viruses)) OR TS=(hepatitis virus type c)) OR TS=(hcv)) OR TS=(h c v)) OR TS=(vhc)) OR TS=(v h c)) OR TS=(hepacivirus)) OR TS=(hepaciviruses)) OR TS=(hcv viral)) OR TS=(hcv infected)) OR TS=(hcv infection)) OR TS=(hcv rna)) OR TS=(hepatitis c virus rna)) OR TS=(parenterally transmitted non a non)) OR TS=(pt-nanbh)

#2 ((((((((((((((((((((((((((((((((((((((((((((TS=(clinical laboratory diagnoses)) OR TS=(clinical laboratory diagnostic)) OR TS=(clinical laboratory techniques)) OR TS=(clinical laboratory testing)) OR TS=(diagnose)) OR TS=(diagnoses)) OR TS=(diagnosis of hcv)) OR TS=(diagnosis)) OR TS=(diagnostic techniques and procedures)) OR TS=(diagnostic)) OR TS=(hcv infection diagnosis)) OR TS=(hcv testing)) OR TS=(mass screening)) OR TS=(mass screenings)) OR TS=(molecular diagnostic techniques)) OR TS=(screening approach)) OR TS=(screening)) OR TS=(testing diagnostic)) OR TS=(plasma levels)) OR TS=(sera)) OR TS=(serum levels)) OR TS=(dried blood filter)) OR TS=(dried blood spot)) OR TS=(dried blood)) OR TS=(dried sample)) OR TS=(filter paper)) OR TS=(Whatman)) OR TS=(DBS)) OR TS=(assay kits)) OR TS=(hcv assays)) OR TS=(hcv pcr assay)) OR TS=(hcv pcr method)) OR TS=(hcv pcr)) OR TS=(hcv rna levels)) OR TS=(hcv rna quantification assays)) OR TS=(hcv rna quantification)) OR TS=(hepatitis c markers)) OR TS=(hepatitis markers)) OR TS=(immunoassay)) OR TS=(quantitative assays)) OR TS=(quantitative reverse transcription pcr)) OR TS=(real time pcr)) OR TS=(rna levels)) OR TS=(roche cobas taqman assays)) OR TS=(roche cobas taqman hcv)

#3 ((((((((((((((((((((((((((TS=(antigens)) OR TS=(cleia method)) OR TS=(core antigen assay)) OR TS=(core antigen assays)) OR TS=(core antigen test)) OR TS=(core antigen)) OR TS=(hcv ag assay)) OR TS=(hcv ag detection)) OR TS=(hcv ag)) OR TS=(hcv antigen testing)) OR TS=(hcv antigen)) OR TS=(hcv core antigen assay)) OR TS=(hcv core antigen assays)) OR TS=(hcv core antigen detection)) OR TS=(hcv core antigen determination)) OR TS=(hcv core antigen testing)) OR TS=(hcv core antigen)) OR TS=(hcv core protein)) OR TS=(hcv core region)) OR TS=(hcv cp)) OR TS=(hcvcoreag)) OR TS=(hcvcag)) OR TS=(hepatitis c antigens)) OR TS=(hepatitis c virus core antigen)) OR TS=(hepatitis c virus core)) OR TS=(hepatitis non a non b antigen)) OR TS=(viral core proteins)

#4 (((((((((((((((((((((((((((((((((((((((TS=(accuracy)) OR TS=(correlation)) OR TS=(correlations)) OR TS=(negative predictive power)) OR TS=(negative predictive value)) OR TS=(negative predictive values)) OR TS=(NPV)) OR TS=(positive predictive power)) OR TS=(positive predictive value)) OR TS=(positive predictive values)) OR TS=(PPV)) OR TS=(receiver operating characteristics)) OR TS=(regression analysis)) OR TS=(ROC)) OR TS=(sensitive)) OR TS=(sensitivities)) OR TS=(sensitivity)) OR TS=(specific)) OR TS=(specificity)) OR TS=(Abbott ARCHITECT HCV Ag assay)) OR TS=(Abbott ARCHITECT HCV Ag test)) OR TS=(Abbott ARCHITECT HCV Antigen assay)) OR TS=(Abbott ARCHITECT i2000SR)) OR TS=(Abbott ARCHITECT test)) OR TS=(Abbott Diagnostics)) OR TS=(Abbott HCV Ag)) OR TS=(Abbott HCV core antigen)) OR TS=(Abbott Laboratories)) OR TS=(ARCHITECT)) OR TS=(ARCHITECT ci8200)) OR TS=(Architect core antigen)) OR TS=(Architect HCV Ag)) OR TS=(ARCHITECT HCV Core antigen)) OR TS=(ARCHITECT HCVAg)) OR TS=(ARCHITECT i2000SR)) OR TS=(ARCHITECT system)) OR TS=(ARCHITECTHCVAg)) OR TS=(ARCHITECT-i2000R)) OR TS=(cleia method)) OR TS=(chemiluminescence immunoassay)

#5 #1 AND #2 AND #3 AND #4

#6 #5 NOT (DT==("REVIEW" OR "MEETING ABSTRACT" OR "PROCEEDINGS PAPER" OR "NOTE" OR "BOOK CHAPTER" OR "EDITORIAL MATERIAL" OR "LETTER"))

## **Search strategy SCOPUS**

( TITLE-ABS-KEY ( "hepatitis c virus" ) OR TITLE-ABS-KEY ( "hepatitis c like virus" ) OR TITLE-ABS-KEY ( {hepatitis virus type c} ) OR TITLE-ABS-KEY ( {hcv} ) OR TITLE-ABS-KEY ( {h c v} ) OR TITLE-ABS-KEY ( {vhc} ) OR TITLE-ABS-KEY ( {v h c} ) OR TITLE-ABS-KEY ( "hepacivirus" ) OR TITLE-ABS-KEY ( {hcv viral} ) OR TITLE-ABS-KEY ( {hcv infected} ) OR TITLE-ABS-KEY ( {hcv infection} ) OR TITLE-ABS-KEY ( "hcv rna" ) OR TITLE-ABS-KEY ( {pt nanbh} ) OR TITLE-ABS-KEY ( {parenterally transmitted non a non} ) ) AND ( TITLE-ABS-KEY ( {clinical laboratory diagnoses} ) OR TITLE-ABS-KEY ( {clinical laboratory techniques} ) OR TITLE-ABS-KEY ( {clinical laboratory testing} ) OR TITLE-ABS-KEY ( "diagnose" ) OR TITLE-ABS-KEY ( {diagnostic techniques and procedures} ) OR TITLE-ABS-KEY ( {hcv infection diagnosis} ) OR TITLE-ABS-KEY ( {hcv testing} ) OR TITLE-ABS-KEY ( "mass screening" ) OR TITLE-ABS-KEY ( {molecular diagnostic techniques} ) OR TITLE-ABS-KEY ( "screening*" ) OR TITLE-ABS-KEY ( {testing diagnostic} ) OR TITLE-ABS-KEY ( {plasma levels} ) OR TITLE-ABS-KEY ( {sera} ) OR TITLE-ABS-KEY ( {serum levels} ) OR TITLE-ABS-KEY ( "dried blood*" ) OR TITLE-ABS-KEY ( "dried sample*" ) OR TITLE-ABS-KEY ( {DBS} ) OR TITLE-ABS-KEY ( {filter paper} ) OR TITLE-ABS-KEY ( {Whatman} ) OR TITLE-ABS-KEY ( {assay kits} ) OR TITLE-ABS-KEY ( "hcv assay" ) OR TITLE-ABS-KEY ( hcv pcr* ) OR TITLE-ABS-KEY ( {hcv rna levels} ) OR TITLE-ABS-KEY ( "hcv rna quantification*" ) OR TITLE-ABS-KEY ( "hepatitis C markers" ) OR TITLE-ABS-KEY ( {immunoassay} ) OR TITLE-ABS-KEY ( "quantitative assay" ) OR TITLE-ABS-KEY ( {quantitative reverse transcription pcr} ) OR TITLE-ABS-KEY ( {real time pcr} ) OR TITLE-ABS-KEY ( {rna levels} ) OR TITLE-ABS-KEY ( {roche cobas taqman} ) ) AND ( TITLE-ABS-KEY ( {antigens} ) OR TITLE-ABS-KEY ( {cleia method} ) OR TITLE-ABS-KEY ( "core antigen*" ) OR TITLE-ABS-KEY ( "hcv ag*" ) OR TITLE-ABS-KEY ( "hcv antigen*" ) OR TITLE-ABS-KEY ( "hcv core antigen*" ) OR TITLE-ABS-KEY ( "hcv core*" ) OR TITLE-ABS-KEY ( "hcv cp" ) OR TITLE-ABS-KEY ( "hcvcag" ) OR TITLE-ABS-KEY ( "hcvcoreag" ) OR TITLE-ABS-KEY ( "hepatitis c antigen" ) OR TITLE-ABS-KEY ( {viral core proteins} ) ) AND ( TITLE-ABS-KEY ( { accuracy } ) OR TITLE-ABS-KEY ( { correlation } ) OR TITLE-ABS-KEY ( { correlations } ) OR TITLE-ABS-KEY ( { negative predictive power } ) OR TITLE-ABS-KEY ( { negative predictive value } ) OR TITLE-ABS-KEY ( { negative predictive values } ) OR TITLE-ABS-KEY ( { NPV} ) OR TITLE-ABS-KEY ( { positive predictive power } ) OR TITLE-ABS-KEY ( { positive predictive value } ) OR TITLE-ABS-KEY ( { positive predictive values } ) OR TITLE-ABS-KEY ( { PPV} ) OR TITLE-ABS-KEY ( { receiver operating characteristics } ) OR TITLE-ABS-KEY ( { regression analysis } ) OR TITLE-ABS-KEY ( { ROC } ) OR TITLE-ABS-KEY ( { sensitive } ) OR TITLE-ABS-KEY ( { sensitivities } ) OR TITLE-ABS-KEY ( { sensitivity } ) OR TITLE-ABS-KEY ( { specific } ) OR TITLE-ABS-KEY ( { specificity } ) OR ALL ( { Abbott ARCHITECT HCV Ag assay } ) OR ALL ( { Abbott ARCHITECT HCV Ag test } ) OR ALL ( { Abbott ARCHITECT HCV Antigen assay } ) OR ALL ( { Abbott ARCHITECT i2000SR } ) OR ALL ( { Abbott ARCHITECT test } ) OR ALL ( { Abbott Diagnostics } ) OR ALL ( { Abbott HCV Ag } ) OR ALL ( { Abbott HCV core antigen } ) OR ALL ( { Abbott Laboratories } ) OR ALL ( { ARCHITECT } ) OR ALL ( { ARCHITECT ci8200} ) OR ALL ( { Architect core antigen } ) OR ALL ( { Architect HCV Ag } ) OR ALL ( { ARCHITECT HCV Core antigen } ) OR ALL ( { ARCHITECT HCVAg } ) OR ALL ( { ARCHITECT i2000SR } ) OR ALL ( { ARCHITECT system } ) OR ALL ( { ARCHITECTHCVAg } ) OR ALL ( { ARCHITECT-i2000R } ) ) AND ( EXCLUDE ( DOCTYPE , "re" ) OR EXCLUDE ( DOCTYPE , "cp" ) OR EXCLUDE ( DOCTYPE , "le" ) OR EXCLUDE ( DOCTYPE , "sh" ) ) AND ( EXCLUDE ( DOCTYPE , "no" ) OR EXCLUDE ( DOCTYPE , "ed" ) OR EXCLUDE ( DOCTYPE , "ch" ) OR EXCLUDE ( DOCTYPE , "dp" ) )

## **Search strategy Cochrane**

#1 MeSH descriptor: [Hepatitis C] explode all trees

#2 MeSH descriptor: [Hepacivirus] explode all trees

#3 ("hepatitis c"):ti,ab,kw

#4 ("hepatitis c virus"):ti,ab,kw

#5 ("hepatitis c viruses"):ti,ab,kw

#6 ("hcv"):ti,ab,kw

#7 ("h c v"):ti,ab,kw

#8 ("vhc"):ti,ab,kw

#9 ("hepacivirus"):ti,ab,kw

#10 ("hcv viral"):ti,ab,kw

#11 ("hcv infected"):ti,ab,kw

#12 ("hcv infection"):ti,ab,kw

#13 ("hcv rna"):ti,ab,kw

#14 ("hepatitis c virus rna"):ti,ab,kw

#15 #1 or #2 or #3 or #4 or #5 or #6 or #7 or #8 or #9 or #10 or #11 or #12 or #13 or #14

#16 MeSH descriptor: [Diagnosis] explode all trees

#17 MeSH descriptor: [Diagnostic Techniques and Procedures] explode all trees

#18 MeSH descriptor: [Clinical Laboratory Techniques] explode all trees

#19 MeSH descriptor: [Mass Screening] explode all trees

#20 MeSH descriptor: [Nucleic Acid Amplification Techniques] explode all trees

#21 MeSH descriptor: [RNA] explode all trees

#22 ("clinical laboratory diagnoses"):ti,ab,kw

#23 ("clinical laboratory diagnostic"):ti,ab,kw

#24 ("clinical laboratory techniques"):ti,ab,kw

#25 ("clinical laboratory testing"):ti,ab,kw

#26 ("diagnose"):ti,ab,kw

#27 ("diagnoses"):ti,ab,kw

#28 ("diagnosis of hcv"):ti,ab,kw

#29 ("diagnosis"):ti,ab,kw

#30 ("diagnostic techniques and procedures"):ti,ab,kw

#31 ("diagnostic"):ti,ab,kw

#32 ("hcv infection diagnosis"):ti,ab,kw

#33 ("hcv testing"):ti,ab,kw

#34 ("mass screening"):ti,ab,kw

#35 ("mass screenings"):ti,ab,kw

#36 ("molecular diagnostic techniques"):ti,ab,kw

#37 ("screening approach"):ti,ab,kw

#38 ("screening"):ti,ab,kw

#39 ("testing diagnostic"):ti,ab,kw

#40 ("plasma levels"):ti,ab,kw

#41 ("sera"):ti,ab,kw

#42 ("serum levels"):ti,ab,kw

#43 ("dried blood"):ti,ab,kw

#44 ("dried sample"):ti,ab,kw

#45 ("filter paper"):ti,ab,kw

#46 ("Whatman"):ti,ab,kw

#47 ("DBS"):ti,ab,kw

#48 ("assay kits"):ti,ab,kw

#49 ("hcv assays"):ti,ab,kw

#50 ("hcv pcr"):ti,ab,kw

#51 ("hcv rna levels"):ti,ab,kw

#52 ("hcv rna quantification"):ti,ab,kw

#53 ("hepatitis markers"):ti,ab,kw

#54 ("immunoassay"):ti,ab,kw

#55 ("quantitative assays"):ti,ab,kw

#56 ("quantitative reverse transcription pcr"):ti,ab,kw

#57 ("real time pcr"):ti,ab,kw

#58 ("rna levels"):ti,ab,kw

#59 ("roche cobas taqman"):ti,ab,kw

#60 #16 or #17 or #18 or #19 or #20 or #21 or #22 or #23 or #24 or #25 or #26 or #27 or #28 or #29 or #30 or #31 or #32 or #33 or #34 or #35 or #36 or #37 or #38 or #39 or #40 or #41 or #42 or #43 or #44 or #45 or #46 or #47 or #48 or #49 or #50 or #51 or #52 or #53 or #54 or #55 or #56 or #57 or #58 or #59

#61 MeSH descriptor: [Hepatitis C Antigens] explode all trees

#62 ("antigens"):ti,ab,kw

#63 ("cleia method"):ti,ab,kw

#64 ("core antigen assays"):ti,ab,kw

#65 ("core antigen"):ti,ab,kw

#66 ("hcv ag"):ti,ab,kw

#67 ("hcvcAg"):ti,ab,kw

#68 ("hcv antigen"):ti,ab,kw

#69 ("hcv core antigen"):ti,ab,kw

#70 ("hcv core protein"):ti,ab,kw

#71 ("hcv core region"):ti,ab,kw

#72 ("hepatitis c antigens"):ti,ab,kw

#73 ("hepatitis c virus core antigen"):ti,ab,kw

#74 ("hepatitis c virus core"):ti,ab,kw

#75 ("viral core proteins"):ti,ab,kw

#76 #61 or #62 or #63 or #64 or #65 or #66 or #67 or #68 or #69 or #70 or #71 or #72 or #73 or #74 or #75

#77 ("accuracy"):ti,ab,kw

#78 ("correlation"):ti,ab,kw

#79 ("correlations"):ti,ab,kw

#80 ("negative predictive power"):ti,ab,kw

#81 ("negative predictive value"):ti,ab,kw

#82 ("negative predictive values"):ti,ab,kw

#83 ("NPV"):ti,ab,kw

#84 ("positive predictive power"):ti,ab,kw

#85 ("positive predictive value"):ti,ab,kw

#86 ("positive predictive values"):ti,ab,kw

#87 ("PPV"):ti,ab,kw

#88 ("receiver operating characteristics"):ti,ab,kw

#89 ("regression analysis"):ti,ab,kw

#90 ("ROC"):ti,ab,kw

#91 ("sensitive"):ti,ab,kw

#92 ("sensitivities"):ti,ab,kw

#93 ("sensitivity"):ti,ab,kw

#94 ("specific"):ti,ab,kw

#95 ("specificity"):ti,ab,kw

#96 "Abbott ARCHITECT i2000SR"

#97 "Abbott Diagnostics"

#98 "Abbott Laboratories"

#99 "ARCHITECT"

#100 "ARCHITECT ci8200"

#101 "ARCHITECT i2000SR"

#102 "ARCHITECT system"

#103 "cleia method"

#104 "chemiluminescence immunoassay"

#105 #77 or #78 or #79 or #80 or #81 or #82 or #83 or #84 or #85 or #86 or #87 or #88 or #89 or #90 or #91 or #92 or #93 or #94 or #95 or #96 or #97 or #98 or #99 or #100 or #101 or #102 or #103 or #104

#106 #15 and #60 and #76 and #105

#
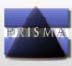
 Additional file 3: Risk of bias assessment adapted from QUADAS-2

## **Domain 1: Patient Selection**

**1.1 Risk of bias: Could the selection of patients have introduced bias?**

**Signaling questions and answer guidelines**

**Signaling question 1**: Was a consecutive or random sample of patients or specimens enrolled?

- Yes: the study enrolled a consecutive or random samples of eligible patients
- No: the study selected patients by selection or convenience
- Unclear: the study did not report how the patient selection was

**Signaling question 2**: Was a case-control design avoided?

- Yes: the study is not a case-control design
- No: the study is a case-control design
- Unclear: the study design was not reported, or we were unable to identify from the text

**Signaling question 3**: Did the study avoid inappropriate exclusions?

- Yes: the study enrolled consecutive or random samples of eligible patients
- No: the study excluded samples based on their prior testing, as these exclusions significantly reduce the generalizability of a study’s findings
- Unclear: the study did not report exclusion criteria, or we were unable to identify from the text

Risk of bias was evaluated as 'low risk' if studies scored 'yes' on all the questions or two questions were answered with 'yes' and one with 'unclear'; 'high risk' if two or more questions were answered with 'no' or one question was answered with 'no' and two with 'unclear'; and 'unclear risk' if studies scored 'unclear' on all the questions, two questions are answered with 'unclear' and one with 'yes', two questions were answered with 'yes' and one with 'no', or each question was answered with 'yes', 'no' and 'unclear'

**1.2 Applicability: Are there concerns that the included patients and setting do not match the review question?**

- Low concern: the study enrolled a broad study population in any setting
- High concern: the study inappropriately included healthy or blood donors only
- Unclear concern: the population was not well characterized, or we could not identify if a study’s patients did not match our review question.

## **Domain 2: Index Test**

**2.1 Risk of bias: Could the conduct or interpretation of the index test have introduced bias?**

**Signaling question 1**: Were the index test results interpreted without knowing the reference standard results?

- Yes: results of the reference standard (HCV-RNA) test were blinded. Studies where the HCVcAg test was reported blinded to the HCV-RNA test or if it was clear that the HCVcAg test was reported before the results of the HCV-RNA test were available
- No: results of reference standard were unblended. The results of the HCVcAg test were reported on previous knowledge of the HCV-RNA test
- Unclear: we were unable to identify whether samples were tested or the HCVcAg test results were interpreted without knowledge of the HCV-RNA test results

**Signaling question 2**: If a threshold was used, was it pre-specified?

- Yes: the limit of detection for commercially available HCVcAg tests was pre-specified by the manufacturer
- No: the threshold of the HCVcAg test was personally selected to optimize sensitivity and specificity, leading to over-optimistic estimates of test performance
- Unclear: we could not determine whether the threshold of the HCVcAg test was pre-specified or not

Risk of bias was evaluated as 'low risk' if studies scored 'yes' on all the questions, or one question was answered with 'yes' and the other one with 'unclear'; 'high risk' if studies scored 'no' on all the questions, or one question was answered with 'no' and another one with 'unclear'; and 'unclear risk' if studies scored 'unclear' on all the questions; or questions were answered with 'yes' and 'no'

**2.2 Applicability: Are there concerns that the index test, its conduct, or interpretation differ from the review question?**

- Low concern: the HCVcAg test was performed according to the manufacturer's recommendations
- High concern: the HCVcAg test procedure was inconsistent with the manufacturer recommendations (i.e., additional processing steps were added), or there was a delayed assessment of samples to perform the HCVcAg test
- Unclear concern: the HCVcAg test was not discussed in the study, or we were unable to determine how the HCVcAg test was conducted or interpreted

## **Domain 3: Reference standard**

**3.1 Risk of bias: Could the reference standard, its conduct, or its interpretation have introduced bias?**

**Signaling question 1**: Is the reference standard likely to classify the target condition correctly?

- Yes: the reference standard for HCV-RNA testing was a nucleic acid amplification test
- No: the reference standard for HCV-RNA testing was not a nucleic acid amplification test, or a combination of different nucleic acid amplification tests was used
- Unclear: there is insufficient information about which was reference standard for HCV-RNA testing used, or we were unable to identify from the text

**Signaling question 2**: Were the reference standard results interpreted without knowing the index test results?

- Yes: studies where the HCV-RNA test was interpreted blindly to the results of the HCVcAg test
- No: studies where the HCV-RNA test was not interpreted blindly to the results of the HCVcAg test
- Unclear: we were unable to identify whether samples were tested or if the HCV-RNA test results were interpreted without knowledge of the HCVcAg test results

**3.2 Applicability: Are there concerns that the target condition as defined by the reference standard does not match the question?**

- Low concern: the HCV-RNA test was performed according to the manufacturer's recommendations
- High concern: the HCV-RNA test procedure was inconsistent with the manufacturer recommendations, or there was a delayed assessment of samples to perform the HCV-RNA test
- Unclear concern: the HCV-RNA test was not discussed in the study, or we were unable to determine how the HCV-RNA test was conducted or interpreted

## **Domain 4: Flow and timing**

**4.1 Risk of bias: Could the patient flow have introduced bias?**

**Signaling question 1**: Was there an appropriate interval between the index test and reference standard?

- Yes: samples for HCVcAg and reference standards tests did obtain at the same time
- No: samples for HCVcAg and reference standards tests did not obtain at the same time
- Unclear: it was not discussed in the study, or we were unable to determine when HCVcAg and reference standards tests were conducted or interpreted

**Signaling question 2**: Did all patients in the study receive the same reference standard?

- Yes: the study used the same rt-PCR for all samples
- No: the study used different types of rt-PCR to analyze all samples
- Unclear: it was not defined in the study, or we were unable to interpret the used rt-PCR

**Signaling question 3**: Were all patients included in the analysis?

- Yes: the whole population recruited into the study was included in the analysis, or any exclusion was adequately described
- No: participants were missing, or the study excluded samples without a given reason
- Unclear: not enough information was given to assess why participants were excluded from the analysis, or we were unable to find an explanation for the exclusion of samples

Risk of Bias was evaluated as 'low risk' if studies scored 'yes' on all the questions or two questions were answered with 'yes' and one with 'unclear'; 'high risk' if two or more questions were answered with 'no' or one question was answered with 'no' and two with 'unclear'; and 'unclear risk' if studies scored 'unclear' on all the questions, two questions are answered with 'unclear' and one with 'yes', two questions were answered with 'yes' and one with 'no', or each question was answered with 'yes', 'no' and 'unclear'

## **Summary of the quality assessment by using QUADAS-2**

|  | **Risk of bias** | | | | **Concerns regarding applicability** | | | |
| --- | --- | --- | --- | --- | --- | --- | --- | --- |
| **Author (year)** | **Patient selection** | **Index test** | **Ref. standard** | **Flow and timing** | | **Patient selection** | **Index test** | **Ref. standard** |
| Mederacke et al. (2012) | L | L | L | UC | | L | L | L |
| Alonso et al. (2017) | L | H | L | L | | L | L | L |
| Duchesne et al. (2017) | UC | L | L | L | | L | L | L |
| Loggi et al. (2017) | L | L | L | UC | | L | L | L |
| Mohamed et al. (2017) | L | L | L | UC | | L | L | L |
| Wasitthankasem et al. (2017) | L | L | L | UC | | L | L | L |
| Alonso et al. (2018) | UC | L | L | L | | L | L | L |
| Ponnuvel et al. (2021) | UC | L | L | L | | L | L | L |
| Sun et al. (2022) | L | L | L | H | | L | H | L |
| Ponnuvel et al. (2023) | UC | L | L | L | | L | L | L |

H= high; L= low; Ref = reference; UC = unclear

# Additional fil
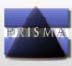
e 4: Figures

## **Figure S1**. **Forest plots of diagnostic accuracy measures for Abbott ARCHITECT HCV Ag assay using a univariate random-effects model.** This figure presents forest plots showing the diagnostic accuracy of the Abbott ARCHITECT HCV Ag assay for detecting active HCV infection in HCV/HBV coinfected individuals compared to a confirmatory nucleic acid test. **Abbreviations**: *95% CI* 95% confidence interval; *df* degrees of freedom; *HBV* hepatitis B virus; *HCV* hepatitis C virus; *I^2^* Higgins' inconsistency index; *Q* Cochran's Q test statistic; *Se* sensitivity.


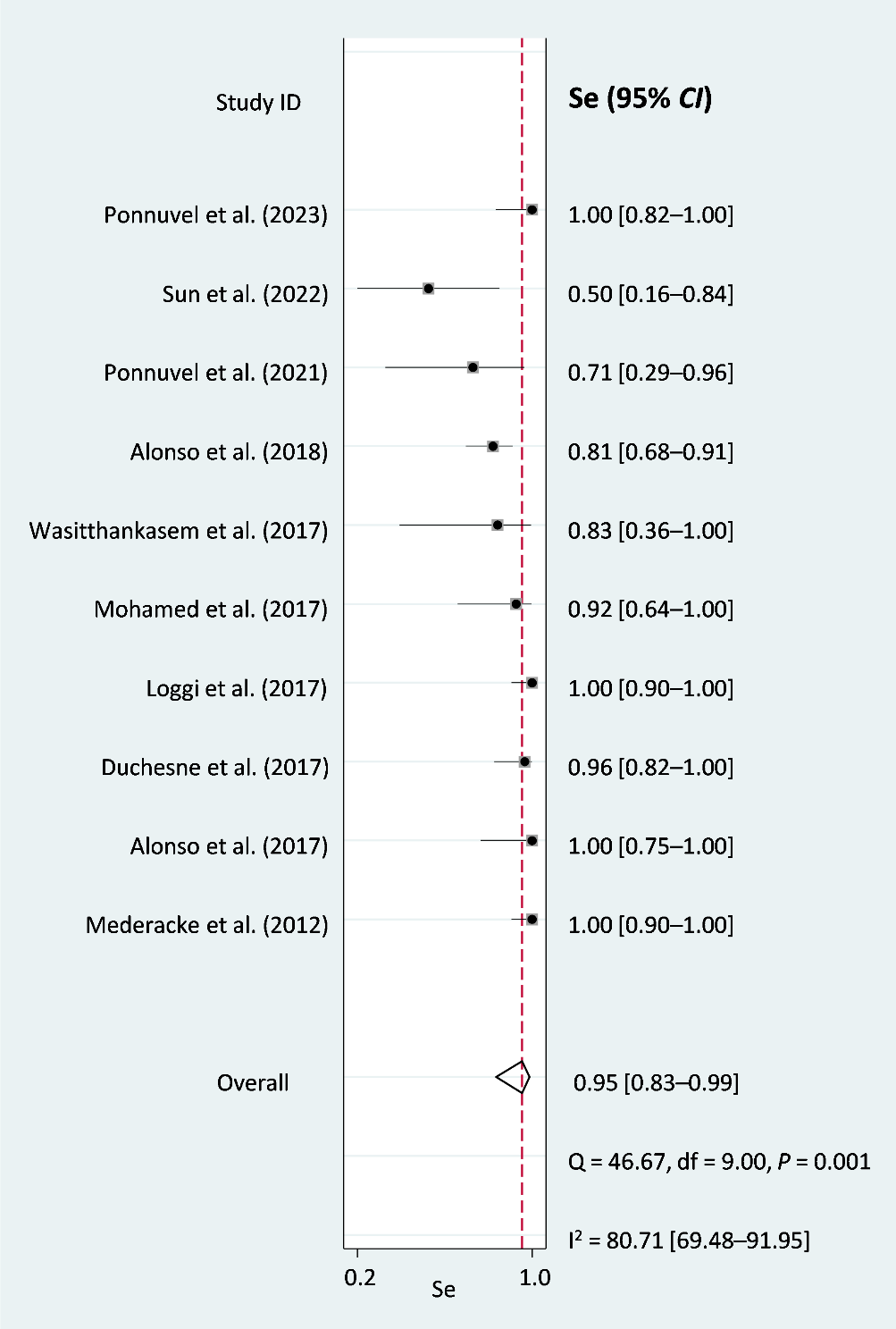


## **Figure S2.** SROC curve plot for the Abbott ARCHITECT HCV Ag assay in detecting active HCV infection in HCV/HBV coinfected individuals compared to a confirmatory nucleic acid test. **Abbreviations**: *95% CI* 95% confidence interval; *AUC* area under the curve; *HCV* hepatitis C virus; *Se* sensitivity; *Sp* specificity; *SROC* summary receiver operating characteristic.


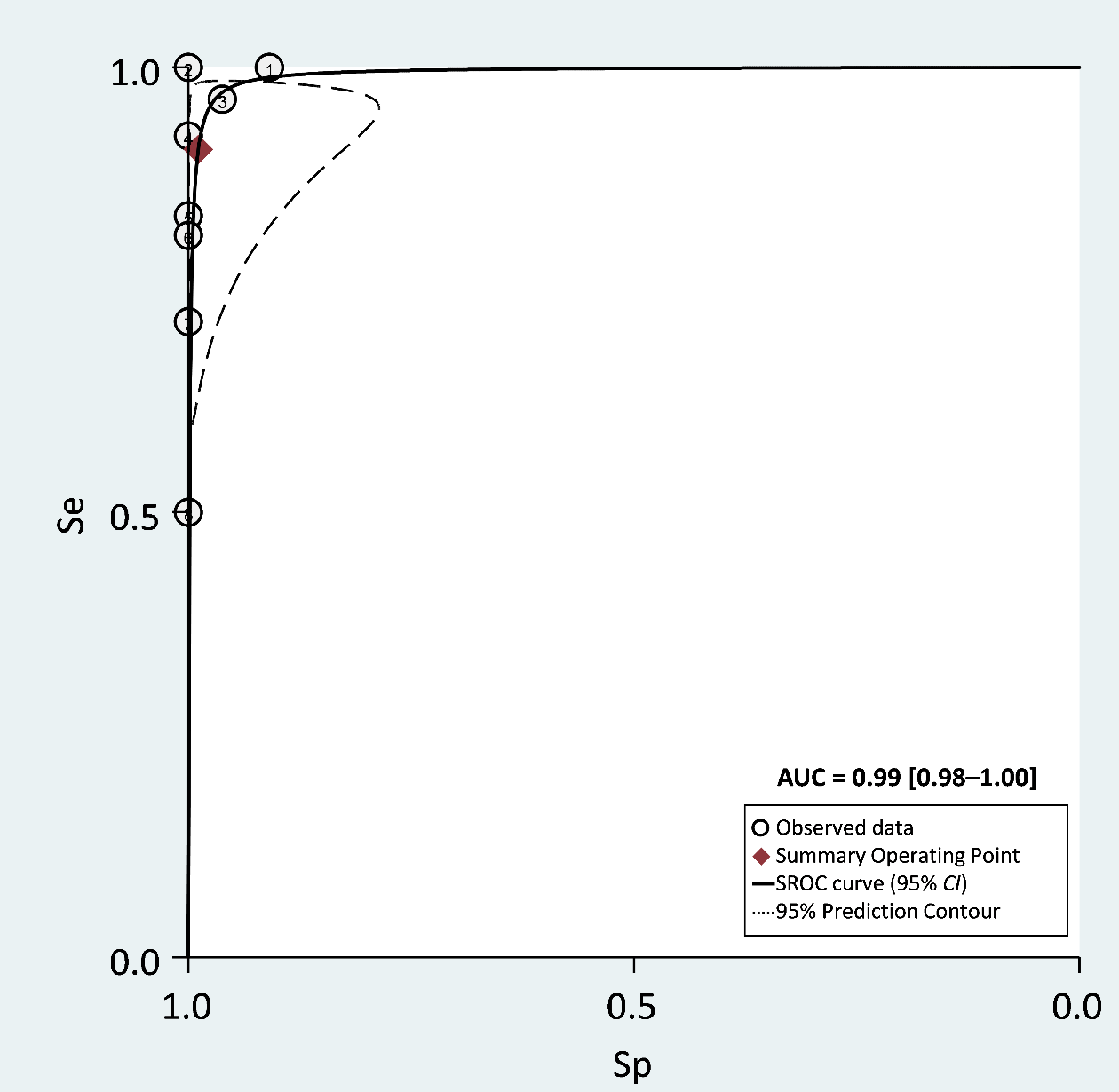


## **Figure S3.** Likelihood ratio scatter plot for the Abbott ARCHITECT HCV Ag assay in detecting active HCV infection in HCV/HBV coinfected individuals compared to a confirmatory nucleic acid test. **Abbreviations**: *95% CI* 95% confidence interval; *HBV* hepatitis B virus; *HCV* hepatitis C virus; *LLQ* left lower quadrant (exclusion only: PLR<10, NLR<0.1); *LUQ* left upper quadrant (confirmation and exclusion: PLR > 10, NLR < 0.1); *NLR* negative likelihood ratio; *PLR* positive likelihood ratio; *RLQ* right lower quadrant (no confirmation or exclusion: PLR < 10, NLR > 0.1); *RUQ* right upper quadrant (confirmation only: PLR > 10, NLR > 0.1)


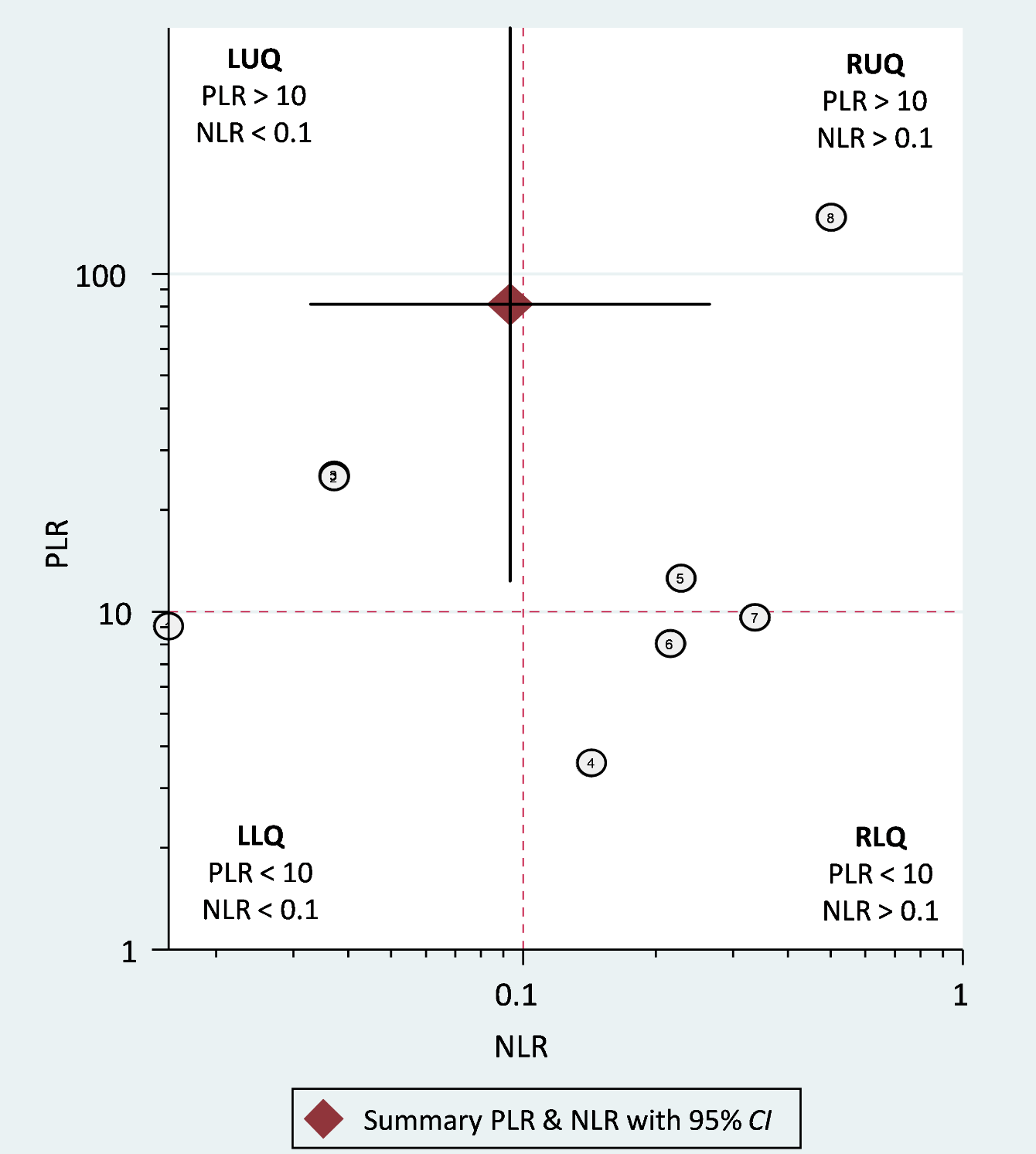


## **Figure S4.** Probability modifying plot for the Abbott ARCHITECT HCV Ag assay in detecting active HCV infection in HCV/HBV coinfected individuals compared to a confirmatory nucleic acid test. **Abbreviations**: *95% CI* 95% confidence interval; *HBV* hepatitis B virus; *HCV* hepatitis C virus; *NLR* negative likelihood ratio; *NPV* negative predictive value; *PLR* positive likelihood ratio; *PPV* positive predictive value; *Post-tp* post-test probability; *Pre-tp* pre-test probability


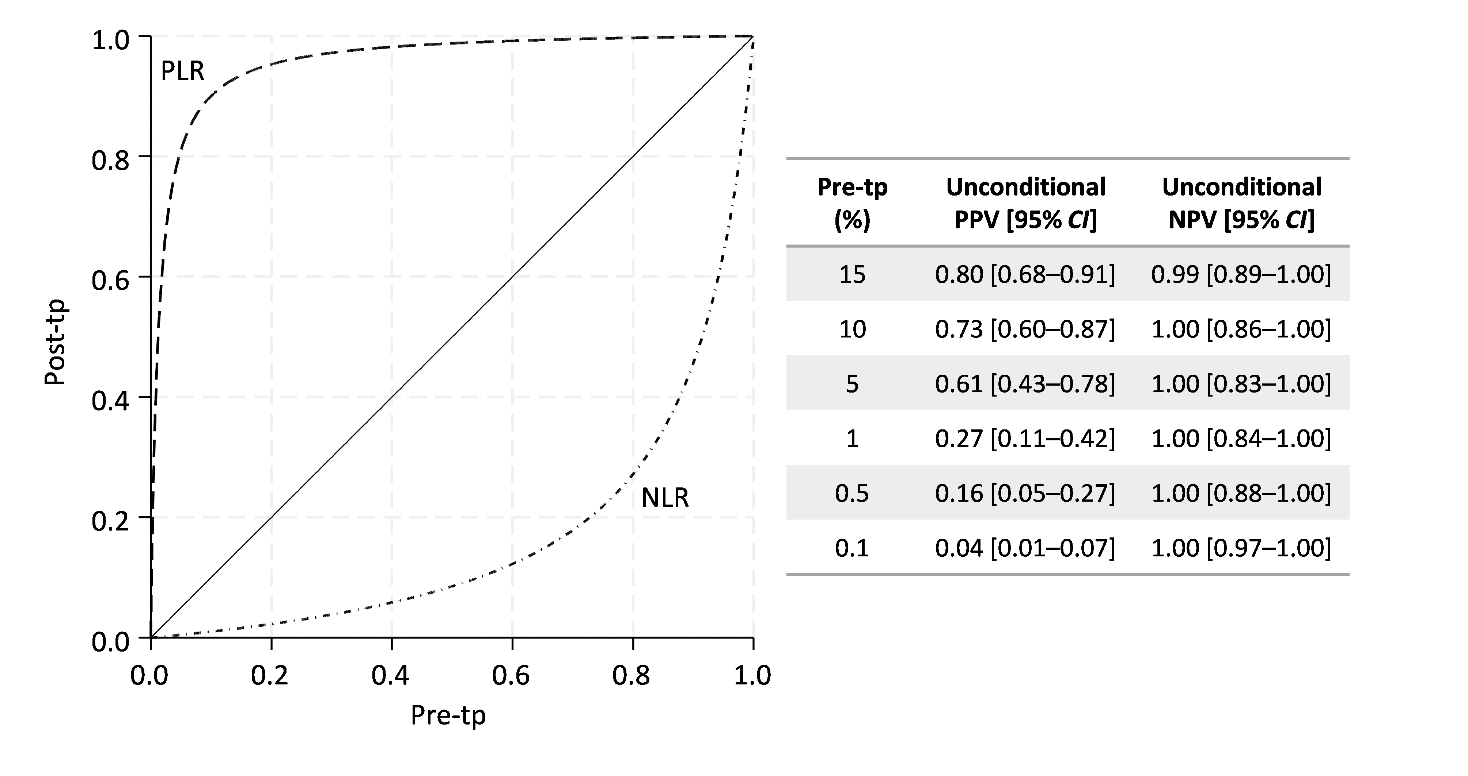


## **Figure S5. Exploration of heterogeneity in the bivariate meta-analysis.** This figure depicts two graphical tools used to explore potential sources of heterogeneity in the bivariate random-effects meta-analysis: **(A)** Galbraith plot and **(B)** bagplot. **Abbreviations**: *DOR* diagnostic odds ratio; *Ln* natural logarithm; *Se* sensitivity; *SE* standard error; *Sp* specificity


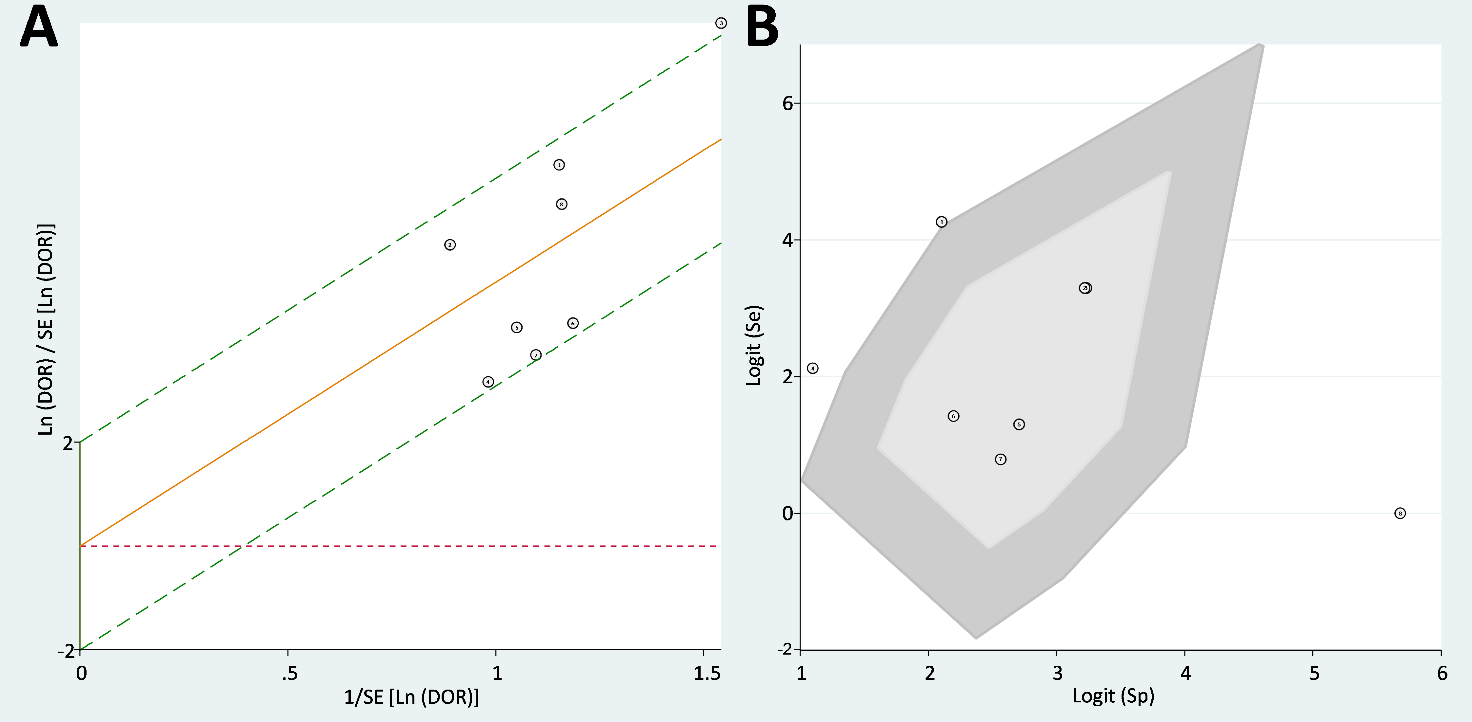


## **Figure S6.** Further exploration of heterogeneity in the bivariate meta-analysis using the Baujat plot


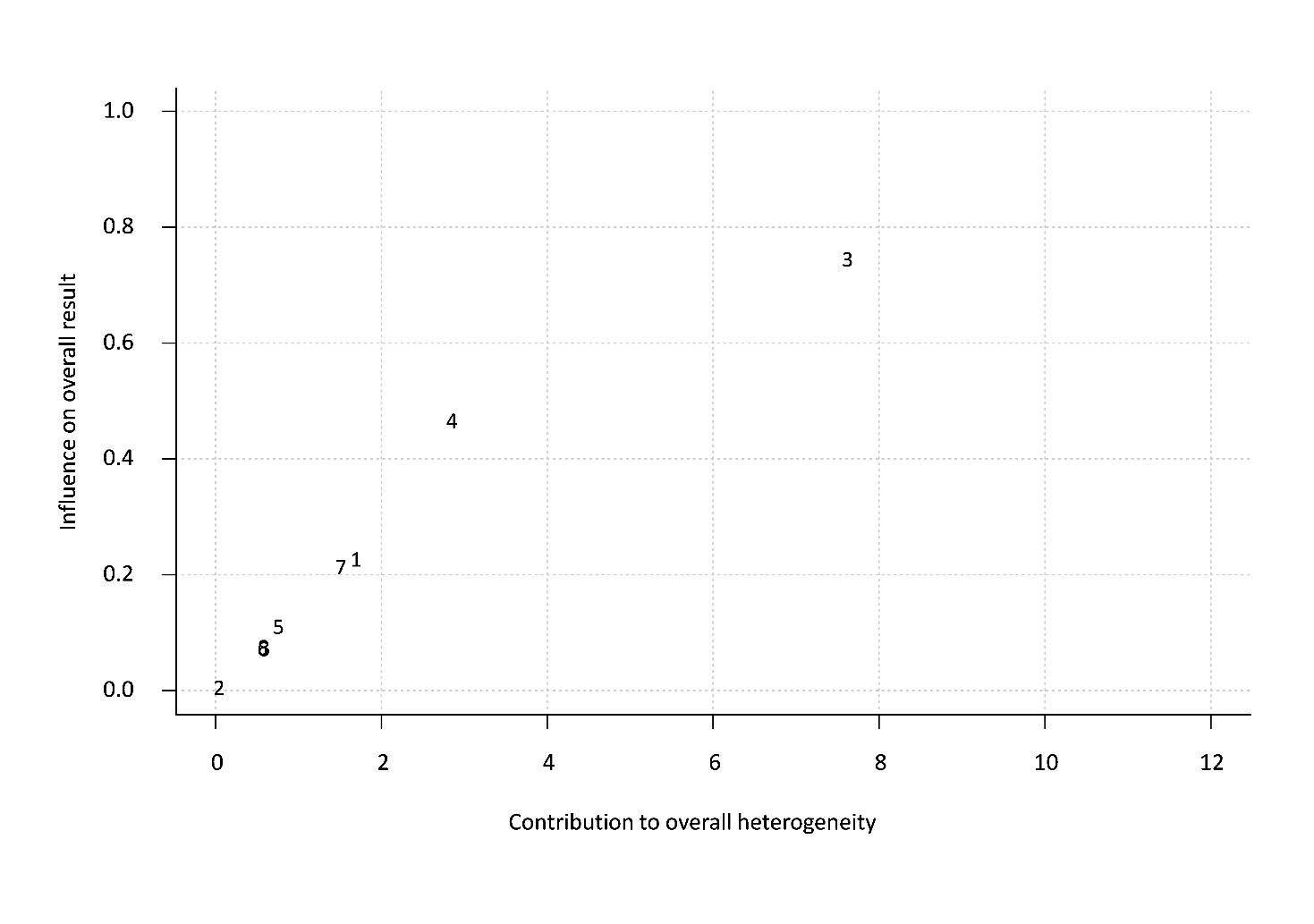


## **Figure S7. (A)** Deeks’ funnel plot and **(B)** Trim and Fill funnel plot for publication bias in the Abbott ARCHITECT HCV Ag assay's ability to detect active HCV infection in HCV/HBV coinfected individuals, compared to a confirmatory nucleic acid test. **Abbreviations**: *DOR* diagnostic odds ratio; *Ln* natural logarithm; *θ_REML_* population effect size estimate obtained using REstricted Maximum Likelihood after applying the Trim and Fill technique to correct for publication bias; *SE* standard error


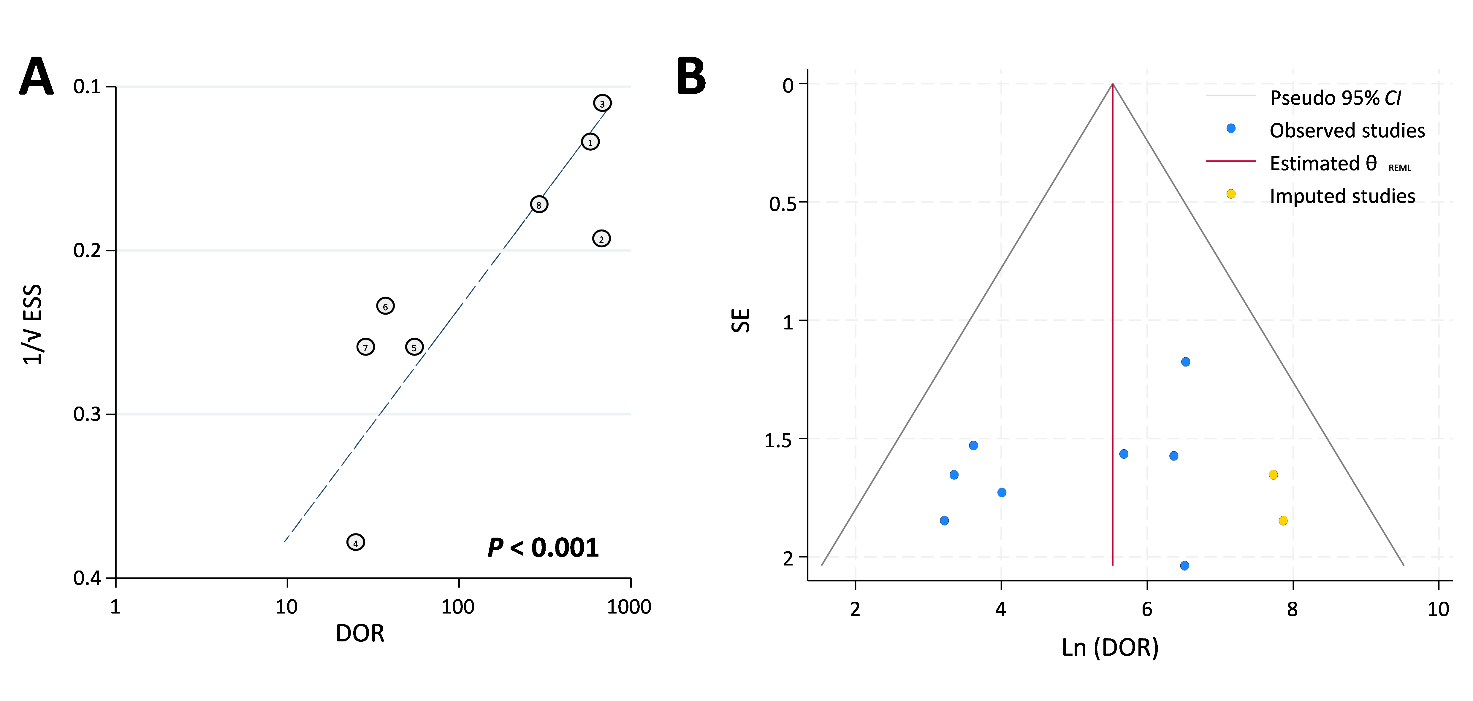


# Additional file 5: Tables

## **Table S1**. Sensitivity analysis for diagnostic performance measures and heterogeneity

| **Deleted study** | **Se [95% *CI*]** | **Sp [95% *CI*]** | **PLR [95% *CI*]** | **NLR [95% *CI*]** | **I^2^ [95% *CI*]** | ***P*-value** |
| --- | --- | --- | --- | --- | --- | --- |
| Mederacke et al. (2012) | 0.87 [0.72–0.94] | 0.99 [0.91–1.00] | 148.1 [8.8–2493.1] | 0.14 [0.06–0.30] | 69 [31–100] | **0.020** |
| Alonso et al. (2017) | 0.89 [0.72–0.96] | 0.99 [0.92–1.00] | 81.0 [11.3–583.3] | 0.11 [0.04–0.31] | 86 [70–100] | **<0.001** |
| Duchesne et al. (2017) | 0.89 [0.70–0.97] | 1.00 [0.80–1.00] | 225.4 [3.7–13570.6] | 0.11 [0.03–0.33] | 84 [66–100] | **0.001** |
| Mohamed et al. (2017) | 0.91 [0.76–0.97] | 0.99 [0.93–1.00] | 81.2 [123–534.4] | 0.09 [0.03–0.27] | 86 [72–100] | **<0.001** |
| Wasitthankasem et al. (2017) | 0.91 [0.72–0.97] | 0.99 [0.93–1.00] | 82.5 [12.2–559.5] | 0.10 [0.03–0.31] | 87 [73–100] | **<0.001** |
| Alonso et al. (2018) | 0.92 [0.74–0.98] | 0.99 [0.92–1.00] | 73.7 [12.0–452.2] | 0.09 [0.03–0.29] | 87 [73–100] | **<0.001** |
| Ponnuvel et al. (2021) | 0.93 [0.75–0.98] | 0.99 [0.92–1.00] | 71.1 [12.0–421.4] | 0.08 [0.02–0.28] | 86 [70–100] | **<0.001** |
| Sun et al. (2022) | 0.92 [0.76–0.98] | 0.99 [0.92–1.00] | 70.0 [12.0–409.0] | 0.08 [0.02–0.26] | 86 [72–100] | **<0.001** |

**Abbreviations**: *95% CI* 95% confidence interval; *I^2^* Higgins' inconsistency index; *NLR* negative likelihood ratio; *PLR* positive likelihood ratio; *Se* sensitivity; *Sp* specificity.

**Table S2**. Results of bivariate meta-regression analysis using Higgins' inconsistency index (I²) for subgroup analysis of the Abbott ARCHITECT HCV Ag assay in detecting active HCV infection in HCV/HBV coinfected individuals compared to a confirmatory nucleic acid test

| **Parameter** | **Category** | **I^2^ [95% *CI*]** | **Х^2^** | ***P*-value** |
| --- | --- | --- | --- | --- |
| Year of publication | Yes: ≤ 2017 | 81 [58–100] | 10.28 | **0.01** |
|  | No: > 2017 |  |  |  |
| LMIC | Yes | 0 [0–100] | 0.17 | 0.92 |
|  | No |  |  |  |
| Sample size | Yes: ≤ 50 | 0 [0–100] | 1.28 | 0.53 |
|  | No: > 50 |  |  |  |
| Biological sample type | Yes: serum | 0 [0–100] | 0.70 | 0.70 |
|  | No: plasma or serum/plasma |  |  |  |
| Sample condition | Yes: frozen | 37 [0–100] | 3.17 | 0.20 |
|  | No: unknown |  |  |  |
| HCV prevalence | Yes: ≤ 50 | 0 [0–100] | 0.36 | 0.83 |
|  | No: > 50 |  |  |  |
| COBAS Ampliprep/COBAS TaqMan HCV Real-time PCR | Yes | 0 [0–100] | 0.27 | 0.87 |
|  | No |  |  |  |
| QUADAS-2 low/unclear overall risk | Yes: Low/unclear | 24 [0–100] | 2.62 | 0.27 |
|  | No: high |  |  |  |
| Low/unclear risk of bias QUADAS-2 | Yes: Low/unclear | 24 [0–100] | 2.62 | 0.27 |
|  | No: high |  |  |  |
| Low/unclear applicability concerns QUADAS-2 | Yes: Low/unclear | 58 [4–100] | 4.72 | **0.09** |
|  | No: high |  |  |  |

**Abbreviations**: *95% CI* 95% confidence interval; *cAg* core antigen; *HBV* hepatitis B virus; *HCV* hepatitis C virus; *IU* international units; *I^2^* Higgins' inconsistency index; *LMIC* low- or middle-income country; *QUADAS-2* Quality Assessment of Diagnostic Accuracy Studies-2; *Х^2^* Pearson's chi-squared test.

## **Table S3**. Results of bivariate meta-regression analysis using sensitivity and specificity for subgroup analysis of the Abbott ARCHITECT HCV Ag assay in detecting active HCV infection in HCV/HBV coinfected individuals compared to a confirmatory nucleic acid test

| **Parameter** | **Category** | **No.** | **Se [95% *CI*]** | ***P*-value** | **Sp [95% *CI*]** | ***P*-value** |
| --- | --- | --- | --- | --- | --- | --- |
| Year of publication | Yes: ≤ 2017 | 5 | 0.96 [0.92–0.99] | 0.11 | 0.96 [0.92–0.99] | **<0.001** |
|  | No: > 2017 | 3 | 0.76 [0.66–0.87] |  | 1.00 [1.00–1.00] |  |
| LMIC | Yes | 4 | 0.91 [0.76–1.00] | 0.77 | 0.99 [0.95–1.00] | 0.11 |
|  | No | 4 | 0.91 [0.78–1.00] |  | 0.99 [0.97–1.00] |  |
| Sample size | Yes: ≤ 50 | 4 | 0.90 [0.77–1.00] | 0.84 | 0.99 [0.96–1.00] | **<0.001** |
|  | No: > 50 | 4 | 0.92 [0.79–1.00] |  | 1.00 [1.00–1.00] |  |
| Biological sample type | Yes: serum | 4 | 0.94 [0.85–1.00] | 0.28 | 0.99 [0.95–1.00] | 0.13 |
|  | No: plasma or serum/plasma | 4 | 0.86 [0.67–1.00] |  | 0.99 [0.97–1.00] |  |
| Sample condition | Yes: frozen | 5 | 0.97 [0.92–1.00] | 0.27 | 0.97 [0.92–1.00] | **<0.001** |
|  | No: unknown | 3 | 0.85 [0.62–1.00] |  | 1.00 [1.00–1.00] |  |
| HCV prevalence | Yes: ≤ 50 | 5 | 0.93 [0.84–1.00] | 0.23 | 0.99 [0.95–1.00] | **0.05** |
|  | No: > 50 | 3 | 0.84 [0.61–1.00] |  | 0.99 [0.97–1.00] |  |
| COBAS Ampliprep/COBAS TaqMan HCV Real-time PCR | Yes | 5 | 0.92 [0.81–1.00] | 0.45 | 0.99 [0.97–1.00] | **0.02** |
|  | No | 3 | 0.89 [0.71–1.00] |  | 0.99 [0.96–1.00] |  |
| QUADAS-2 low/unclear overall risk | Yes: Low/unclear | 6 | 0.92 [0.83–1.00] | 0.23 | 0.97 [0.93–1.00] | **<0.001** |
|  | No: high | 2 | 0.85 [0.56–1.00] |  | 1.00 [1.00–1.00] |  |
| Low/unclear risk of bias QUADAS-2 | Yes: Low/unclear | 6 | 0.92 [0.83–1.00] | 0.23 | 0.97 [0.93–1.00] | **<0.001** |
|  | No: high | 2 | 0.85 [0.56–1.00] |  | 1.00 [1.00–1.00] |  |
| Low/unclear applicability concerns QUADAS-2 | Yes: Low/unclear | 7 | 0.93 [0.86–1.00] | **0.01** | 0.97 [0.93–1.00] | **<0.001** |
|  | No: high | 1 | 0.50 [0.05–1.00] |  | 1.00 [1.00–1.00] |  |

**Abbreviations**: *95% CI* 95% confidence interval; *cAg* core antigen; *HBV* hepatitis B virus; *HCV* hepatitis C virus; *LMIC* low- or middle-income country; *No.* number of articles; *QUADAS-2* Quality Assessment of Diagnostic Accuracy Studies-2; *Se* sensitivity; *Sp* specificity
